# The key mechanism of pulsed electric field in inhibiting the flavor deterioration of whole egg liquid: Targeted regulation of chemical oxidation and microbial succession

**DOI:** 10.1016/j.fochx.2026.104558

**Published:** 2026-10-01

**Authors:** Liu Dong, Enhui Ma, Renrui Zheng, Jun Jiang, Xinqing Lu, Lili Zhang, Songyi Lin

**Affiliations:** a National Engineering Research Center of Seafood, School of Food Science and Technology, Dalian Polytechnic University, Dalian 116034, PR China; b Engineering Research Center of Special Dietary Food of Liaoning Province, Food Engineering Technology Research Center of Liaoning Province, Dalian 116034, PR China; c Liaoning Provincial Professional Technology Innovation Center of Meat Processing and Quality-Safety Control, College of Food and Health, Jinzhou Medical University, Jinzhou 121000, PR China; d Jilin Jinyi Egg Products Co., Ltd., Liaoyuan 136200, PR China; e Dalian Dingtong Technology Development Co., Ltd., Dalian 116081, PR China

**Keywords:** Pulsed electric field, Flavor deterioration, Chemical oxidation, Microbial succession, Whole egg liquid

## Abstract

This study elucidated mechanisms of pulsed electric field (PEF, 30 kV/cm, 100 Hz) and pasteurization (65 °C, 4 min) in inhibiting flavor deterioration of whole egg liquid. PEF extended shelf life to 36 days versus 20 days for pasteurization, with stable physicochemical property (*P* < 0.05). GC-IMS identified 90 compounds. By combined VIP > 1 and ROAV ≥0.1 screening, 2-isopropyl-3-methoxypyrazine, 2-pentylfuran, 2-pentanone, 1-octen-3-one, and (E)-2-nonenal were determined as five key markers. PEF inhibited off-flavor by blocking oxidation and retaining water, with free sulfhydryl loss at 36.53% and bound water at 7.58%, significantly better than pasteurization. 16S rRNA sequencing revealed PEF reduced *Pseudomonas* from 99.0% to 48.2%, reshaping microbial community toward diversity and balance. Pearson and Mantel tests identified *Pseudomonas* and *Shewanella* as key spoilage genera, positively correlated with oxidation and off-flavor compounds (*r* > 0.5, *P* < 0.05). This study provides critical support for preservation of high-quality liquid eggs.

## Highlights

• PEF extended WEL shelf life to 36 days with stability superior to pasteurization.
• GC-IMS and VIP/ROAV identified five key flavor markers driving flavor deterioration.
• PEF blocked oxidation and maintained moisture, suppressing off-flavor formation.
• PEF broke the spoilage bacteria monopoly, reshaping microbial diversity balance.
• Correlations confirmed inhibiting spoilage bacteria prevents flavor deterioration.

## 1 Introduction

Whole egg liquid (WEL) has become a widely used intermediate raw material in modern food industry due to its excellent processability, convenience, and nutritional value (Dong, Lu, et al., 2026). However, WEL is rich in high-quality proteins, unsaturated fatty acids, and various trace elements, providing a suitable substrate for the growth of microorganisms(Dong, Ma, et al., 2026). During refrigerated storage, microbial proliferation can lead to undesirable flavors such as rancidity, fishiness, and ammonia odor, severely limiting product shelf life and quality stability (Gram et al., 1990; Wang et al., 2025). Traditional pasteurization (65 °C, 4 min) is currently the most commonly used microbial inactivation technology in liquid egg production, which can effectively ensure the microbial safety of products (Dong, Lu, et al., 2026). Heat treatment exerts significant negative impacts on egg liquid quality. Studies have shown that thermal processing initiates chain reactions of lipid oxidation, promoting the formation of volatile off-flavor compounds such as aldehydes and ketones (Zhou et al., 2025). Ren et al. (2022) reported that heat treatment exacerbates the generation of undesirable volatile organic compounds (VOCs) in salted egg yolks. Additionally, heat treatment can degrade endogenous antioxidants and natural flavor precursors in egg liquid, resulting in a loss of the mild aroma characteristic of fresh egg products (Sun et al., 2025). It is worth noting that certain dominant spoilage microorganisms possess cold adaptation and sublethal damage repair capabilities, making thermal treatment often insufficient to completely prevent their revival during the later stages of storage, ultimately triggering a secondary deterioration of flavor driven by microbial metabolism (Ding et al., 2024; Zheng et al., 2025). From this, it can be seen that the flavor deterioration of the whole egg liquid during storage is the result of the initial oxidative damage induced by heat treatment and the microbial metabolism-driven process during storage. Flavor as the primary sensory attribute determining product commercial value, faces significant challenges in maintaining stability during egg liquid processing. Therefore, developing a novel processing technology capable of overcoming the limitations of thermal treatment by blocking physicochemical and microbial degradation pathways at the source has become an urgent challenge in the egg product industry.

Pulsed electric field (PEF) technology as a non-thermal processing method has recently attracted considerable attention in the liquid egg processing industry. PEF induces irreversible electroporation in microbial cell membranes by applying high-voltage, short-duration pulses to food materials, thereby achieving cold pasteurization (Conde et al., 2024). Compared with thermal sterilization, PEF operates at lower temperatures and requires less energy input, effectively preserving the functional properties and natural flavor of egg liquids (Dong, Ma, et al., 2026). Rios-Corripio et al. (2022) found that PEF-treated pomegranate fermented beverages significantly outperformed thermally processed controls in retaining volatile flavor compounds. Bai et al. (2021) also reported that PEF pretreatment of rice could modulate the metabolic balance of flavor compounds, reducing aldehyde content while enhancing ester-related aromatic components. Pina-Pérez et al. (2009) demonstrated that PEF treatment of skimmed whole egg beverage achieved up to 3 log reductions in microbial inactivation under conditions of 30 kV/cm. Aguiló-Aguayo et al. (2009) further observed that PEF treatment of strawberry juice (35 kV/cm) preserved levels of aromatic VOCs and suppressed the increase of off-flavor VOCs. Dong, Lu, et al. (2026) found that, PEF-treated samples showed a significant reduction in microbial counts and improved flavor when the electric field strength was 30 kV/cm compared to pasteurized samples. This indicates that PEF has been proven to synergistically enhance both safety and flavor quality.

Currently, various analytical methods have been developed in the field of food flavor analysis. Among them, gas chromatography-ion mobility spectrometry (GC-IMS) as an emerging technique for analyzing volatile compounds, demonstrates significant advantages over traditional gas chromatography–mass spectrometry (GC–MS) in terms of response speed and detection capability for trace substances (Sun et al., 2025). Owing to its high resolution and rapid separation characteristics, this technology has been extensively applied in the dynamic monitoring of volatile flavor components in food systems. Particularly during short-term storage, subtle changes often occur in food flavor and the ultra-high sensitivity of GC-IMS enables reliable identification and quantification of such low-abundance VOCs (Zhang et al., 2024). Recently, Song et al. (2025) further confirmed that GC-IMS can effectively track the evolution of volatile flavor profiles in egg yolk powder during storage, providing strong technical support for assessing product quality stability. Dong, Lu, et al. (2026) employed GC-IMS technology to evaluate the effects of PEF and pasteurization on the flavor quality of WEL, demonstrating that PEF effectively inactivates microorganisms while preserving the suppression of off-flavor development during storage. Dong et al. (2024) revealed the relationship between characteristic VOCs and structure in egg powder based on GC-IMS technology, confirming the feasibility of GC-IMS in flavor characterization.

However, existing studies have primarily focused on immediate microbial inactivation effects or changes in single quality parameters in WEL, lacking an in-depth analysis of the underlying mechanisms behind flavor deterioration during storage. Therefore, this study investigated the regulatory mechanisms of pasteurization and PEF treatment on the flavor quality of WEL during storage. Based on GC-IMS technology, the evolution patterns of VOCs in WEL during storage were tracked, and the key flavor VOCs were screened. Combined with the 16S rRNA high throughput sequencing technology, the diversity evolution patterns of the core spoilage causing communities were revealed. And the dynamic indicators of protein oxidation, lipid peroxidation, and water migration were simultaneously monitored. By constructing a multivariate correlation network, the mechanism by which PEF inhibits flavor deterioration by targeting the reshaping of the microecological balance and blocking the chemical oxidation chain reaction was clarified. This research provides theoretical basis and technical support for the precise control of off-flavor VOCs in the processing of high-quality liquid egg products.

## 2 Materials and methods

### 2.1 Materials and reagents

The WEL used in this experiment was prepared from fresh eggs purchased from Dalian Qianhe Agricultural Market (Dalian, China). Plate count agar medium (HB0101) was provided by Qingdao HaiBo Biotechnology Co., Ltd. (Qingdao, China). Carbon standards (C4-C9) were supplied by Sinopharm Chemical Reagent Co., Ltd. (Beijing, China). All chemical reagents were of analytical grade.

### 2.2 Preparation and treatment of WEL

The preparation of experimental samples followed the method reported by Dong, Lu, et al. (2026). Fresh eggs were surface-washed and disinfected, then cracked aseptically under clean benches to collect the liquid. The WEL was stirred and high-pressure homogenized. The sample solution was collected and aliquoted into sterile sampling bags, then stored at 4 °C for 5–6 h to simulate temporary storage conditions in industrial production. Based on the previous research, the parameters for pasteurization and PEF treatment were as follows (Dong, Lu, et al., 2026; Dong, Ma, et al., 2026). Pasteurization was performed using a constant temperature water bath system. WEL samples were aliquoted into sterile conical flasks and fully submerged in a preheated 65 °C water bath. Timing began once the center temperature of the sample reached and stabilized at 65 °C, and the samples were held at this temperature for 4 min. PEF treatment was conducted according to the method described by Dong, Ma, et al. (2026), using PEF experimental machine to treat WEL samples. The electric field strength was set at 30 kV/cm, pulse frequency at 100 Hz, pulse width at 4 μs, and flow rate at 1.5 mL/s. A square wave voltage waveform was applied, and the actual voltage between electrodes was monitored in real time via a digital oscilloscope. Sample inlet and outlet temperatures were measured using digital thermometers, with the inlet temperature strictly maintained at 4 °C. The maximum temperature rise throughout the process was limited to no more than 5 °C to eliminate thermal effects. All sample aliquoting and transfer operations were carried out within clean benches, and the samples were immediately stored at 4 °C.

### 2.3 Microbial count

The total plate count of the WEL was determined according to the method reported by Dong, Lu, et al. (2026). Briefly, 1 mL of the sample was serially diluted tenfold with 0.85% sterile physiological saline and shaken thoroughly to mix. Subsequently, 100 μL of the appropriately diluted sample solution was evenly spread onto the surface of plate count agar (PCA) medium using a sterile spreader. The inoculated petri dishes were then inverted and incubated in a constant temperature incubator at 36 ± 1 °C for 48 ± 2 h. After incubation, plates with colony counts ranging from 30 to 300 CFU/plate were selected for enumeration. All experiments were conducted with three parallel replicates. The microbial inactivation effect was expressed as the logarithmic value (Log₁₀ N), where N represents the number of culturable microorganisms in the sample (CFU/mL).

### 2.4 Determination of pH value

The pH value of WEL was determined according to the method reported by Han et al. (2025). 100 mL of sample was taken and three parallel samples were set. The pH value of each sample was measured at a constant temperature of 25 °C using a calibrated digital pH meter (PB-10, Sartorius Scientific Instruments Co., Ltd., Beijing, China).

### 2.5 Determination of chromaticity

The Chromaticity of WEL samples were determined using a colorimeter (HunterLab, UltraScan VIS, USA). The test conditions were set as follows: the standard illuminant was D65 light source, the measurement aperture was 4 mm, and each sample was measured three times. The results were expressed in the CIE L* a* b* color space coordinates, and the average value of the three measurements was taken. Among them, L* represents the sample's lightness, a* represents the chromaticity component in the red-green direction (positive value for red, negative value for green), and b* represents the chromaticity component in the yellow-blue direction (positive value for yellow, negative value for blue) (Dong, Gao, et al., 2026).

### 2.6 Identification and analysis of VOCs based on GC-IMS technology

The VOCs in the WEL samples were qualitatively and semi-quantitatively analyzed using a fully automatic flavor analyzer (FlavourSpec®, G.A.S., Dortmund, Germany). The method was slightly modified based on the description by Song et al. (2025). Specifically, 3 mL of WEL sample was placed in a 20 mL headspace vial, sealed, and incubated at 60 °C with constant shaking (500 rpm) for 20 min. The GC inlet temperature was set at 80 °C, and the headspace gas was automatically injected with a volume of 800 μL. The total analysis time was 32 min. The chromatographic separation was performed using a DB-WAX capillary column, with the column temperature maintained at 60 °C. High-purity nitrogen gas was used as the carrier gas, and the flow rate program was set as follows: 2 mL/min from 0 to 2 min, linearly increasing to 10 mL/min from 2 to 12 min, and maintaining at 150 mL/min from 12 to 32 min. The IMS drift tube was purged with a constant flow of 150 mL/min of nitrogen gas, and the temperature was controlled at 45 °C to ensure a stable ion migration environment. To ensure the accuracy of qualitative identification, this study employed external carbon standard compounds (N-ketones C_4_ to C_9_) and utilized their retention time (RT) and drift time (DT) data to establish a standard curve. The NIST2024 gas chromatography retention index (RI) database and G.A.S. IMS drift time (DT) database in the Vocal software were used for qualitative analysis of VOCs in WEL. The RI matching tolerance was set at ±4%, and the DT matching tolerance was set at ±3% to ensure the reliability and specificity of compound identification. Since the aim of this study is to explore the dynamic differences of volatile components, a relative quantitative strategy was adopted. The peak volume data were extracted by the Laboratory Analysis Viewer (LAV) software and used as a relative content indicator. The Reporter plugin was used to generate two-dimensional (2D) spectrogram, three-dimensional (3D) topographic maps, and difference comparison maps. The Gallery Plot plugin was further utilized to construct a fingerprint gallery plot, enabling visual analysis and comparison of the dynamic evolution of VOC composition across groups.

### 2.7 Determination of free sulfhydryl content

The free sulfhydryl content was determined using the Ellman reagent method (Sheng et al., 2024). 1 mL of sample was added to 10 mL Tris-Gly buffer, and then 1 mL of this diluted solution was mixed with 10 μL of a 4 mg/mL DTNB solution. The mixture was vortexed thoroughly and incubated at 25 °C in the dark for 15 min. A blank control was prepared by replacing DTNB with an equal volume of buffer. Absorbance was measured at 412 nm. The concentration of free sulfhydryl groups was calculated according to the following formula:

$$\text{Free sulfhydryl content}\;\left(\mu \mathrm{mol}/g\;\text{protein}\right)=\frac{73.53\times A_{412}\times D}{C}$$

where A_412_ is the absorbance value; D is the dilution factor; C is the protein concentration of the sample (mg/mL); and 73.53 is the extinction coefficient conversion constant.

### 2.8 Determination of total volatile basic nitrogen content

According to the method of Zheng et al. (2025), total volatile basic nitrogen (TVB-N) content determined using an automatic azotometer. A 10 mL sample of WEL was mixed with 90 mL deionized water and shaken at 4 °C for 10 min. Then, 5 mL of the filtrate was transferred into a digestion tube, followed by the addition of 5 mL of 10 g/L magnesium oxide solution, and distillation was carried out for 5 min. The instrument automatically completed alkalization, distillation, and titration. The content of TVB-N was calculated according to the following formula:

$$\mathrm{TVB}-N\;\left(\mathrm{mg}/100g\right)=\frac{\left(V_{1}-V_{2}\right)\times c\times 14}{m}\times 100$$

Where: V_1_ and V_2_ are the volumes (mL) of standard hydrochloric acid solution consumed by the sample and blank, respectively; C is the concentration of the standard hydrochloric acid solution (mol/L); m is the mass of the sample (g); 14 is the mass (mg) of nitrogen equivalent to 1 mL of standard hydrochloric acid titration solution.

### 2.9 Determination of peroxide value

The method described by Zhang et al. (2025) was followed. The sample was mixed with petroleum ether and left to stand for 12 h for extraction. After filtration and rotary evaporation, 0.5 g of the obtained oil sample was dissolved in 30 mL of a mixture of trichloromethane and glacial acetic acid. Then, 1 mL of saturated potassium iodide solution was added, thoroughly shaken and allowed to stand in the dark for 3 min. The solution was titrated with a standard sodium thiosulfate solution using starch as an indicator. The peroxide value (POV) was calculated using the following formula:

$$\mathrm{POV}\;\left(m\mathrm{mol}/kg\right)=\frac{\left(V-V_{0}\right)\times c\times 1000}{2\times m}$$

where V and V₀ are the volumes (mL) of standard sodium thiosulfate solution consumed by the sample and blank, respectively; C is the concentration (mol/L) of the standard sodium thiosulfate solution; and m is the mass (g) of the sample.

### 2.10 Determination of malondialdehyde content

Determination of lipid oxidation level in WEL by the thiobarbituric acid reactive substance method (Lu et al., 2024). Accurately weigh 2 mL of sample into a centrifuge tube, add 10 mL of 10% trichloroacetic acid solution containing 0.1% EDTA, and vortex for 30 min to extract. Add 2 mL of the sample to 2 mL of 0.02 mol/L thiobarbituric acid reagent. Incubate the mixture at 100 °C for 40 min. Cool to ambient temperature, then centrifuge at 8000 r/min for 15 min at 4 °C. Recover the clear supernatant and record its absorbance at 450 nm, 532 nm, and 600 nm (A_450_, A_532_, A_600_). MDA concentration is determined using the following equation:

$$\mathrm{MDA}\;\left(\mathrm{\mu mol}/\mathrm{kg}\right)=\frac{5\times \left(12.9\left(A532-A600\right)-2.58\times A450\right)}{w}$$

where w is the sample mass (g).

### 2.11 Determination of the moisture distribution state

The distribution characteristics of water in different physical states and their migration behavior during storage in WEL were determined using the low-field nuclear magnetic resonance (LF-NMR) analysis method established by Sheng et al. (2024). Accurately pipette 5 mL of sample into a standard LF-NMR glass sample vial, ensuring no air bubbles remain. The transverse relaxation decay signals were collected using the CPMG (Carr-Purcell-Meiboom-Gill) pulse sequence. Instrument parameters were set as follows: main magnetic field strength 0.5 T, proton resonance frequency 21.7 MHz; 90° radiofrequency pulse width 14 μs, 180° radiofrequency pulse width 28 μs; echo time 0.5 ms; repetition time 2000 ms; number of echoes 3000; number of data points 1024; number of accumulated scans 16. The relaxation decay curves were fitted and inverted using MultiExp Inv software to obtain the T₂ relaxation spectrum. Based on relaxation kinetic characteristics, water components were classified into three categories: bound water (T_21_, 0–10 ms), immobile water (T_22_, 10–100 ms), and free water (T_23_, 100–1000 ms). The percentage of peak area corresponding to each component relative to the total peak area represents its relative content (%).

### 2.12 16 s RNA high-throughput sequencing analysis

After the experiment, WEL samples were collected from each treatment group at the end of storage under sterile conditions. The samples were immediately placed into sterile sampling bags and transported on dry ice to the laboratory. The procedure was slightly modified based on the method described by Wang et al. (2025). Total genomic DNA was extracted from the samples using the E.Z.N.A.® Soil DNA Kit. A 0.5 g sample was mixed with magnetic beads and lysis buffer, then thoroughly disrupted by vortexing in a bead mill (45 Hz) for 250 s. Total DNA was obtained through centrifugation, adsorption, washing, and elution steps. DNA concentration and purity were measured using a NanoDrop 2000 UV spectrophotometer, and DNA integrity was assessed via 1% agarose gel electrophoresis. To prepare for high-throughput sequencing, amplification and library construction were performed targeting the highly variable V3–V4 region of the bacterial 16S rRNA gene. The primers used for amplification are universal specific primer pairs: forward primer 338F and reverse primer 806R. The PCR procedure is as follows: 95 °C for pre-denaturation for 3 min; then 30 cycles of amplification are carried out, each cycle including 95 °C denaturation for 30 s, 55 °C annealing for 30 s, and 72 °C extension for 45 s; finally, the final extension at 72 °C is completed for 10 min. Amplified products were verified by 2% agarose gel electrophoresis and purified using a commercial DNA gel extraction kit. The concentration of purified products was quantified using a Qubit 4.0 fluorometer. Based on the required sequencing depth for each sample, equimolar amounts of purified DNA were pooled to construct paired-end sequencing libraries. Library preparation was carried out using the NEXTFLEX Rapid DNA-Seq Kit, including adapter ligation, magnetic bead size selection, and limited-cycle PCR enrichment. All libraries were sequenced by Shanghai Meiji Biotechnology Co., Ltd. on two platforms: Illumina MiSeq (PE300) and NovoSeq (PE250), for high-throughput sequencing.

### 2.13 Statistical analysis

All bioinformatics analyses were performed on the Majorbio Cloud Platform (https://cloud.majorbio.com). Alpha diversity indices were calculated using the mothur software, and inter-group differences were assessed via one-way analysis of variance (ANOVA), followed by multiple comparisons using Duncan's post-hoc test. Pearson correlation analysis (*R* > 0.5, *P* < 0.05) was employed to examine associations between core microbial genera and physicochemical parameters as well as characteristic VOCs in WEL. And a correlation network was visualized using Chiplot (https://www.chiplot.online). Statistical analyses were conducted with IBM SPSS Statistics 26 (SPSS Inc., Chicago, USA), with significance set at *P* < 0.05. All experiments included at least three replicates, and data are presented as mean ± standard error. Data visualization was performed using Origin 2025 (OriginLab Corporation, MA, USA) and GraphPad Prism 10.1.2 (GraphPad Software, La Jolla, CA, USA).

## 3 Results and discussion

### 3.1 Effects of different treatment methods on microbial growth, pH value and color degree of WEL during storage

The total plate count is a key indicator for evaluating the microbiological safety and shelf-life stability of WEL. As shown in Fig. 1A, the untreated control group (WEL) exhibited an exponential growth trend in colony count during storage. By the fourth day, the total plate count had reached 4.71 Log CFU/mL, far exceeding the spoilage threshold commonly used in food microbiology (Dong, Ma, et al., 2026). In contrast, both the pasteurization treatment group (PAS) and the pulsed electric field treatment group (PEF) significantly inhibited microbial growth. The total plate count in the PAS group reached 4.71 Log CFU/mL on the 20th day, reaching the spoilage threshold. However, the PEF group demonstrated a more persistent antibacterial effect, with the total plate count remaining at 4.70 Log CFU/g until the 36th day of storage, without exceeding the spoilage threshold. Heat treatment may induce the selective enrichment of heat-resistant microorganisms and cause sublethal damage to some microorganisms. These damaged microorganisms have the potential to recover and resuscitate under subsequent refrigeration conditions. In contrast, PEF treatment induced irreversible electroporation effects on the bacterial cell membrane, disrupting the transmembrane potential and ion homeostasis, thereby irreversibly inhibiting microbial metabolic activity and proliferation ability, and thus providing a more long-lasting microbial inhibition effect (Lytras et al., 2024).

**Fig. 1:**
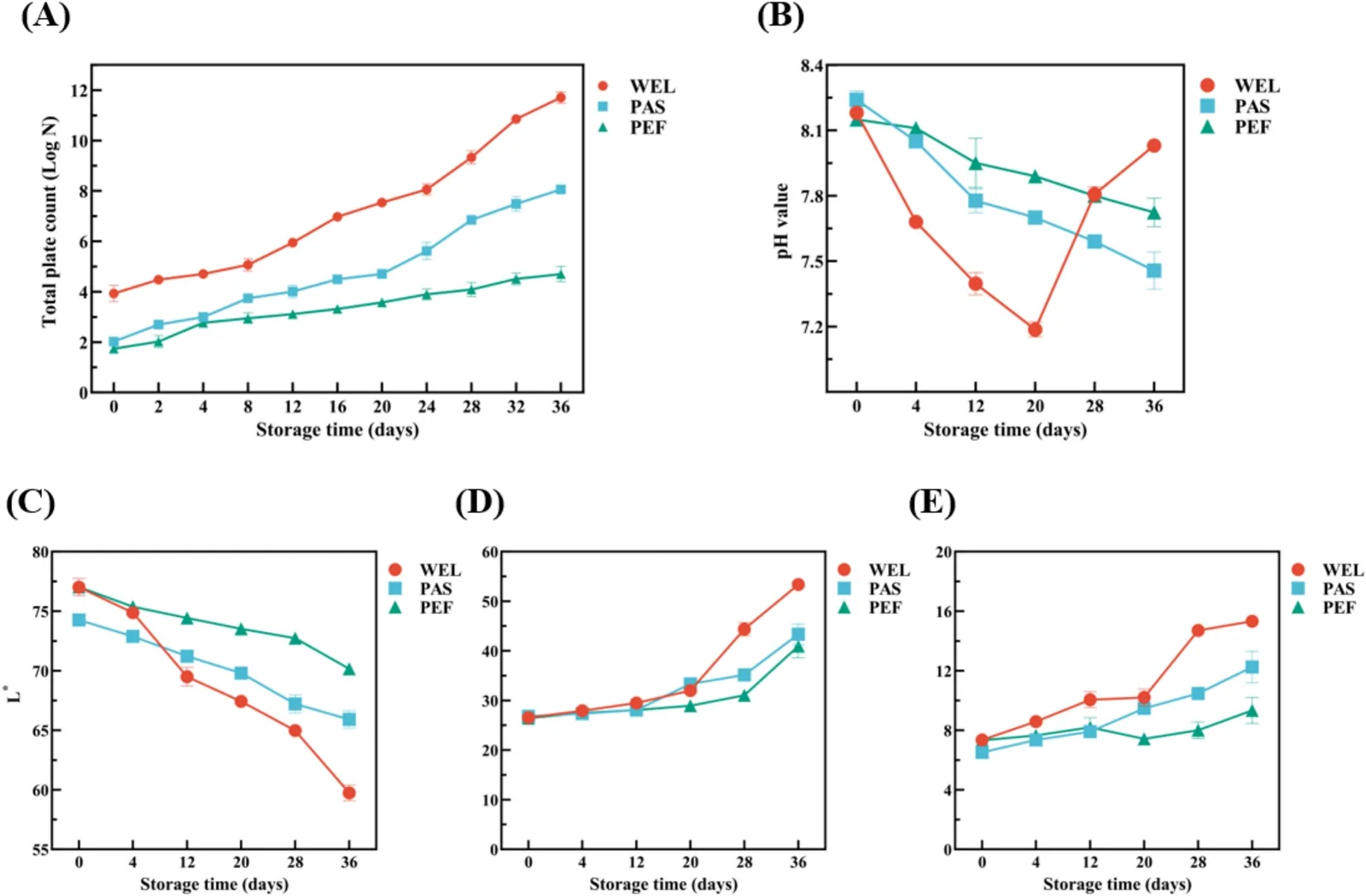
The effects of different treatment methods on the quality of WEL during storage. (A) Total plate count, (B) pH value, (C-E) Color difference parameters.

The pH value changes at key time points during storage were subsequently monitored. As shown in Fig. 1B, the pH value of the WEL control group significantly decreased within the first 20 days of storage, which could be attributed to the accumulation of organic acids due to microbial acid production. Thereafter, the pH value increased, possibly due to the protein hydrolysis and deamination reactions mediated by the dominant spoilage bacteria, which released alkaline nitrogen-containing metabolites (Bi et al., 2025). In contrast, the PEF treatment group showed a small fluctuation in pH value throughout the storage period and remained at a relatively stable level overall, indicating that PEF treatment effectively inhibited the activity of microorganisms and their related metabolic processes, thereby delaying the deterioration of the physicochemical quality of WEL.

In addition, chromaticity parameters (L*, a*, b*) as intuitive physicochemical indicators reflecting the quality deterioration of the samples (Fig. 1C-1E). The L* values of all treatment groups decreased, while the a* and b* values continuously increased. This was indicated that non-enzymatic browning reactions and pigment oxidation degradation occurred in the samples (Alamprese et al., 2019; Dong, Gao, et al., 2026). Among them, the color deterioration of the WEL group was the most significant. Notably, the PEF group demonstrated better color retention ability than the PAS group, with its L* value remaining significantly higher than that of the PAS group throughout the storage period, and the growth rates of a* and b* values were significantly lower. The above results confirmed that PEF, as a non-thermal processing technology, has a distinct advantage in maintaining the physical quality of egg liquid by minimizing heat-induced and oxidation-driven color degradation.

### 3.2 Dynamic changes of VOCs in WEL during storage under different treatment methods

This study employed GC-IMS technology to construct a macroscopic visual map of the VOCs in the flavor deterioration of WEL. The two-dimensional spectrogram (Fig. 2A) and the three-dimensional topographic map (Fig. 2B) visually presented the dynamic evolution of VOCs in WEL under different treatment methods during storage. The VOCs spectrum of the WEL group changed most significantly. Starting from the 4th day of storage, the signal intensity of most characteristic peaks continuously increased, and multiple new characteristic peaks appeared successively; by the 20th day, the overall complexity of the spectrum had significantly increased; and by the 36th day, its contour shape had changed significantly compared to the initial state (day 0). This temporal evolution trend was highly consistent with the growth of microbial populations and the significant deterioration of color in the control group, indicating that microbial metabolic activities were the core driving force for the evolution of the VOCs profile (Han et al., 2025). The spectrum changes in the PAS group were relatively moderate, but a large number of new characteristic peaks appeared from the 12th day of storage, which was consistent with the kinetic characteristics of the gradual recovery of the microbial population in the middle and late stages, further confirming that microbial activity had a decisive regulatory effect on the generation of VOCs. In contrast, the evolution of the spectrum in the PEF treatment group was the most stable. During the storage period from 20 to 36 days, the increase in the number and intensity of characteristic peaks was significantly lower than that of the other two groups. This result was consistent with the report by Yu et al. (2025) that the abundance of volatile compounds in egg yolk powder increases over time during storage. The results indicated that PEF treatment effectively inhibited microbial growth, delayed their metabolic activity, and thus substantially blocked the evolution process of the VOCs profile mediated by microorganisms. In addition, the difference comparison map (Fig. 2C) was normalized based on the samples of each group on day 0, further highlighting the preservation advantages of PEF treatment. The PAS group and the untreated WEL group showed densely distributed red difference signals in the later storage period (day 28 and day 36), indicating that they had accumulated a large amount of newly generated flavor substances. However, the PEF treatment group showed significantly sparser red signals, and the overall spectrum was closer to the initial state, reflecting that it better maintained the original flavor profile of fresh egg liquid.

**Fig. 2:**
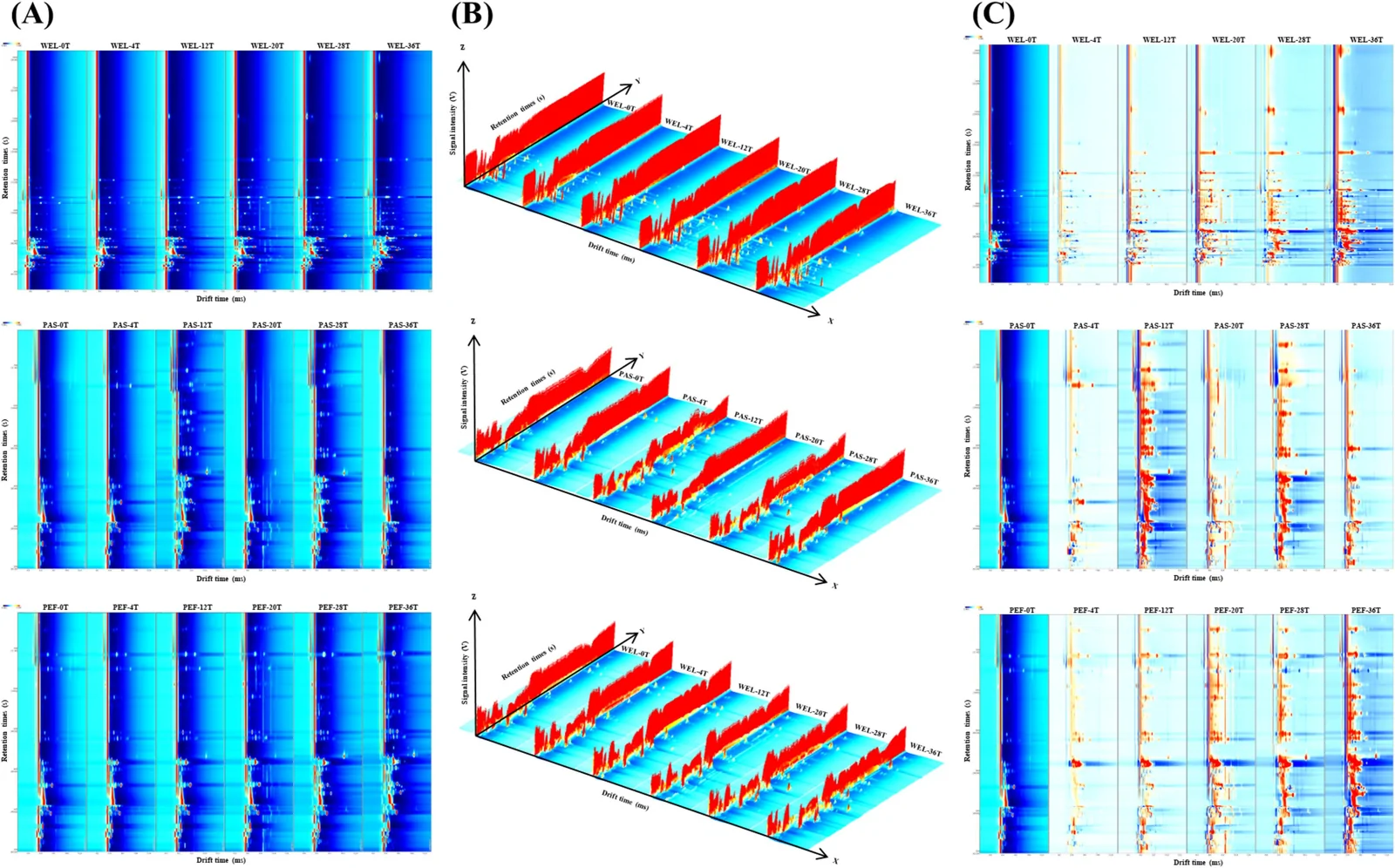

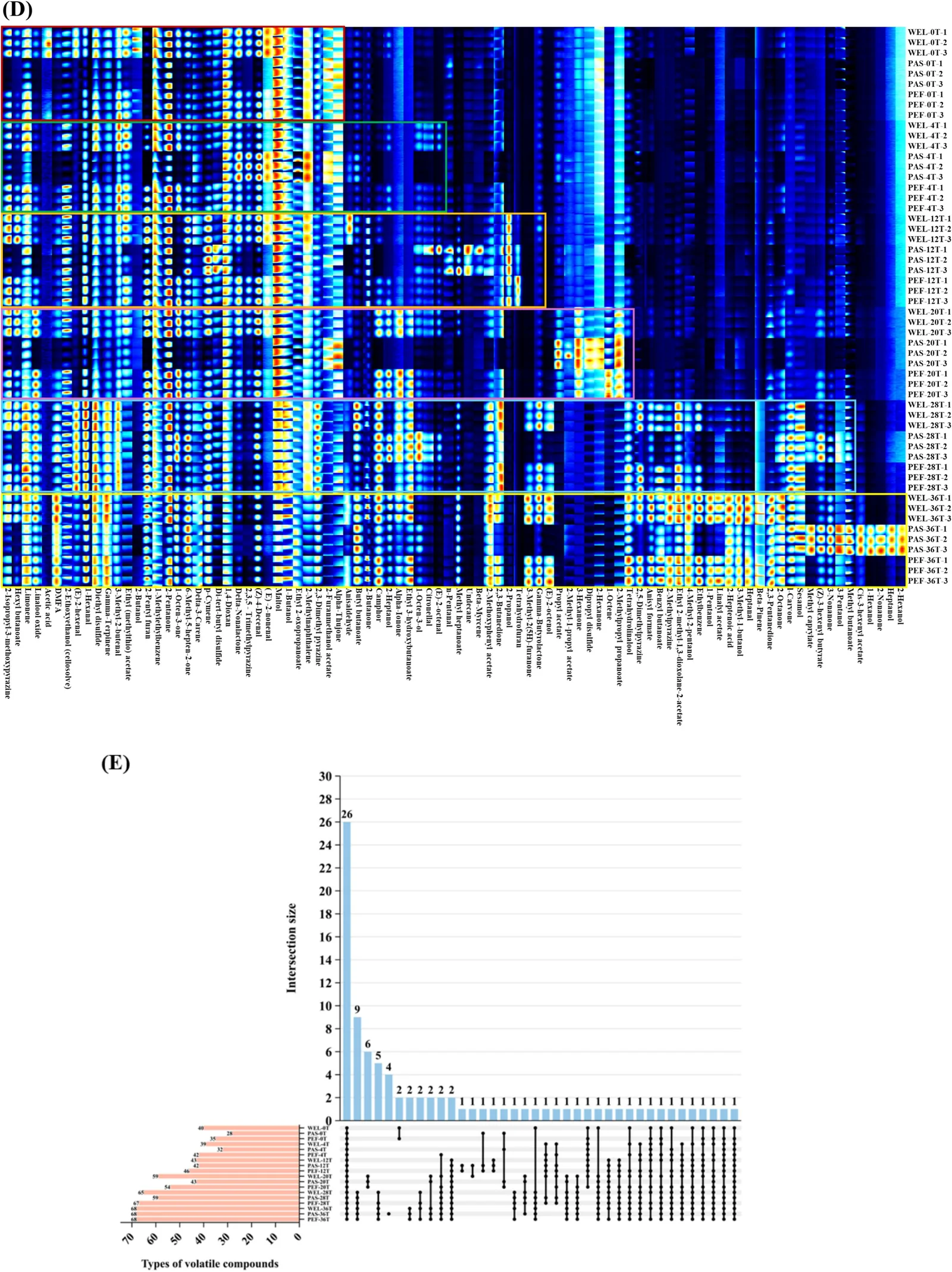

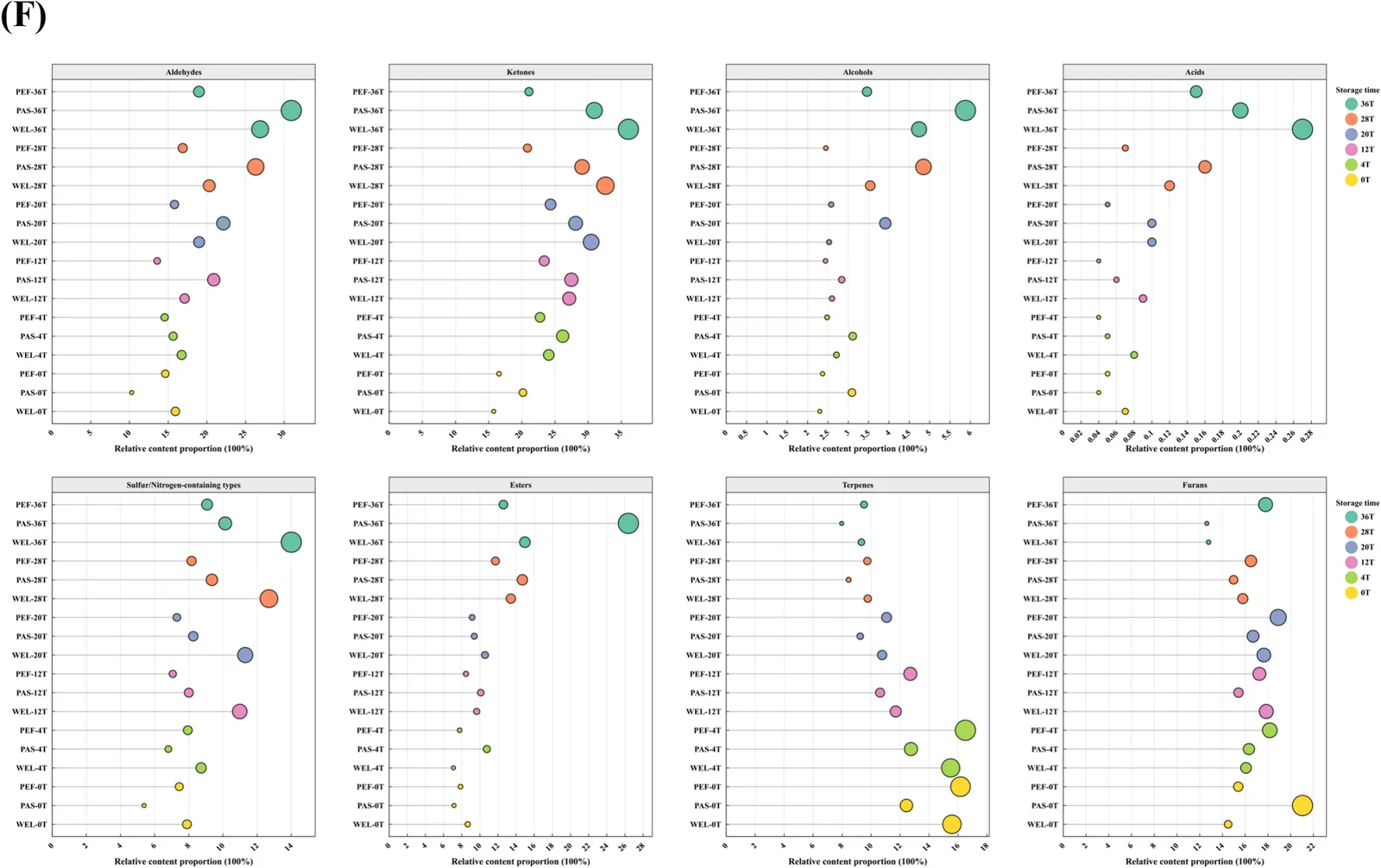
Dynamic changes of VOCs in WEL during storage under different treatments methods. (A) Two-dimensional spectrogram, (B) Three-dimensional topographic map, (C) Difference comparison plot, (D) Characteristic fingerprint gallery plot, (E) Upset plot of distribution VOCs, (F) Relative content of various VOCs.

To further identify the specific flavor VOCs, a characteristic fingerprint gallery plot of WEL was constructed (Fig. 2D). Each row corresponds to an independent sample at a specific storage time point, and each column represents a VOC that has been qualitatively confirmed. The signal intensity was visually characterized by a gradient color scale, with blue to red corresponding to relatively low to high compound concentrations (Song et al., 2025). A total of 90 VOCs were identified in this study (Table 1), covering major chemical categories such as aldehydes, ketones, alcohols, acids, sulfur/nitrogen-containing compounds, esters, terpenes and furans. The key regions marked by rectangular boxes in the figure clearly present the VOC compound clusters at different storage stages. In the early stage of storage, the composition characteristics of VOCs were mainly dominated by the compound clusters on the left side of the red and green boxes in the spectrum. The results showed that the thermal effect of PAS treatment led to changes in some heat-sensitive VOCs at the initial stage. Chen et al. (2025) also reported that thermal treatment intensified the transformation and volatilization of odor compounds in salted egg yolks, resulting in undesirable flavors such as rancidity and off-odors in the final product. However, the samples treated with PEF maintained a highly homologous spectrum with fresh WEL. This indicated that the non-thermal characteristics of PEF effectively preserved the natural flavors in the raw materials. This finding is highly consistent with the research of Rios-Corripio et al. (2022), which showed that PEF treatment of a pomegranate fermented beverage significantly outperformed thermal treatment in terms of volatile flavor retention. At 12 days of storage, characteristic VOCs clusters appeared in the chromatograms of both the WEL control group and the PAS treatment group, and their signal intensities significantly increased. This phenomenon can be attributed to the secondary volatile products derived from lipid oxidation and protein hydrolysis, including short-chain aldehydes, ketones, branched-chain fatty acids, and sulfur-containing small molecules (Dong, Ma, et al., 2026; Zhou et al., 2025). In contrast, PEF treatment significantly inhibited the formation and accumulation of such VOC clusters related to late-stage deterioration. In the later stage of storage, the chromatogram of WEL group showed dense red signals in the right side of the blue and yellow framed areas, indicating a sharp accumulation of off-flavor markers. These compounds mainly originated from lipid oxidation (generating rancid aldehydes and ketones) and microbial degradation of proteins and amino acids (producing organic acids, sulfur-containing compounds, and nitrogen-containing compounds), leading to typical off-flavors such as rancid, sour, and rotten egg odors (Dong, Ma, et al., 2026; Ren et al., 2022; Zhou et al., 2025). Although PAS treatment initially delayed the deterioration, it could not prevent the massive generation of off-flavor substances by the 36th day. In contrast, the PEF group had a much cleaner chromatogram in this area, with strong signals corresponding to sour, rancid, and putrid odors significantly suppressed. This indicated that PEF more effectively maintained the flavor quality stability of WEL.

**Table 1:** Identification of volatile flavor compounds in WEL during storage under different treatment methods based on GC-IMS technology.

Number	Volatile compounds	CAS#	Formula	MW	RI	RT	DT
1	2-Isopropyl-3-methoxypyrazine	25,773–40-4	C8H12N2O	152.2	1414.5	685.69	1.2422
2	Hexyl butanoate	2639-63-6	C10H20O2	172.3	1414.7	686.05	1.4983
3	Limonene	138–86-3	C10H16	136.2	1192.4	362.51	1.2212
4	Linalool oxide	60,047–17-8	C10H18O2	170.3	1444.5	749.75	1.2597
5	Acetic acid	641–97-0	C2H4O2	60.1	1473.5	817.09	1.1452
6	DMFA	681–22-0	C3H7NO	73.1	1360.3	583.74	1.252
7	2-Ethoxyethanol (cellosolve)	110–80-5	C4H10O2	90.1	1249.8	424.4	1.1172
8	(E)-2-hexenal	6728-26-3	C6H10O	98.1	1218.6	389.59	1.1827
9	1-Hexanal	66–25-1	C6H12O	100.2	1095.1	302.39	1.2488
10	Diethyl disulfide	110–81-6	C4H10S2	122.2	1205.3	375.58	1.1375
11	Gamma-Terpinene	99–85-4	C10H16	136.2	1231.5	403.58	1.2212
12	3-Methyl-2-butenal	107–86-8	C5H8O	84.1	1219.4	390.39	1.0921
13	Ethyl (methylthio) acetate	4455-13-4	C5H10O2S	134.2	1443.2	746.84	1.1763
14	2-Butanol	78–92-2	C4H10O	74.1	981.9	250.61	1.3259
15	2-Pentyl furan	3777-69-3	C9H14O	138.2	1248.5	422.93	1.2631
16	1-Methylethylbenzene	98–82-8	C9H12	120.2	1167.8	346.15	1.1371
17	2-Pentanone	107–87-9	C5H10O	86.1	1003.5	262.4	1.1134
18	1-Octen-3-one	4312-99-6	C8H14O	126.2	1293.8	479.05	1.2709
19	6-Methyl-5-hepten-2-one	110–93-0	C8H14O	126.2	1360.1	583.28	1.179
20	Delta-3-Carene	13,466–78-9	C10H16	136.2	1110.6	311.01	1.2249
21	p-Cymene	99–87-6	C10H14	134.2	1303.2	492.55	1.1687
22	Di-tert-butyl disulfide	110–06-5	C8H18S2	178.4	1107.7	309.32	1.3242
23	1,4-Dioxan	123–91-1	C4H8O2	88.1	1066.1	289.1	1.096
24	Delta-Nonalactone	3301-94-8	C9H16O2	156.2	1396.3	649.65	1.2577
25	2,3,5 - Trimethylpyrazine	14,667–55-1	C7H10N2	122.2	1396.2	649.48	1.6314
26	(Z)-4-Decenal	21,662–09-9	C10H18O	154.3	1191.5	361.82	1.3686
27	(E)-2-nonenal	18,829–56-6	C9H16O	140.2	1540.8	998.09	1.4096
28	Maltol	118–71-8	C6H6O3	126.1	1123.4	318.54	1.1493
29	1-Butanol	71–36-3	C4H10O	74.1	1114	313.02	1.1809
30	Ethyl 2-oxopropanoate	617–35–6	C5H8O3	116.1	1235.7	408.26	1.1514
31	2-Methylnaphthalene	91–57-6	C11H10	142.2	1290.6	474.74	1.1915
32	2,3-Dimethyl pyrazine	5910-89-4	C6H8N2	108.1	918.7	216.28	1.1223
33	2-Furanmethanol acetate	623–17-6	C7H8O3	140.1	998.1	260.2	1.4276
34	Alpha-Thujone	546–80-5	C10H16O	152.2	1105.5	308.05	1.8627
35	Anisaldehyde	123–11-5	C8H8O2	136.1	1330.7	534.5	1.2135
36	Butyl butanoate	109–21-7	C8H16O2	144.2	1231.6	403.71	1.3291
37	2-Butanone	78–93-3	C4H8O	72.1	919.8	216.86	1.072
38	Camphor	76–22-2	C10H16O	152.2	1518.1	933.14	1.3265
39	2-Heptanol	543–49-7	C7H16O	116.2	1334.4	540.48	1.7474
40	Alpha-Ionone	127–41-3	C13H20O	192.3	1444.5	749.75	1.5099
41	Ethyl 3-hydroxybutanoate	5405-41-4	C6H12O3	132.2	1517.5	931.21	1.6434
42	1-Octen-3-ol	3391-86-4	C8H16O	128.2	1466.3	799.98	1.1549
43	Citronellal	106–23-0	C10H18O	154.3	1466.8	801.07	1.217
44	(E)-2-octenal	2548-87-0	C8H14O	126.2	1466.2	799.62	1.3295
45	n-Pentanal	110–62-3	C5H10O	86.1	985.2	252.57	1.1961
46	Methyl heptanoate	106–73-0	C8H16O2	144.2	1303.3	492.74	1.3692
47	Undecane	1120-21-4	C11H24	156.3	1107.9	309.44	1.3997
48	Beta-Myrcene	123–35-3	C10H16	136.2	985.6	252.8	1.28
49	2-Methoxyphenyl acetate	613–70-7	C9H10O3	166.2	1304.6	494.69	1.2454
50	2,3-Butanedione	431–03-8	C4H6O2	86.1	978.5	248.64	1.0619
51	2-Propanol	676–30-0	C3H8O	60.1	911.5	212.7	1.2312
52	Tetrahydrofuran	109–99-9	C4H8O	72.1	620.3	107.9	1.068
53	3-Methyl-2(5H)-furanone	22,122–36-7	C5H6O2	98.1	1699.8	1601.3	1.1043
54	Gamma-Butyrolactone	964–80-0	C4H6O2	86.1	1606.7	1214.2	1.0599
55	(E)-2-octenol	18,409–17-1	C8H16O	128.2	1607.3	1216.1	1.1565
56	Propyl acetate	109–60-4	C5H10O2	102.1	980.1	249.57	1.4746
57	2-Methyl-1-propyl acetate	110–19-0	C6H12O2	116.2	1013.4	266.44	1.2252
58	3-Hexanone	589–38-8	C6H12O	100.2	1038.9	277.19	1.4735
59	Dipropyl disulfide	629–19-6	C6H14S2	150.3	1102.2	306.2	1.4729
60	2-Hexanone	591–78-6	C6H12O	100.2	1073.5	292.45	1.5317
61	1-Octene	111–66-0	C8H16	112.2	889.8	202.18	1.4727
62	2-Methylpropyl propanoate	540–42-1	C7H14O2	130.2	1066.1	289.1	1.6916
63	Tetrahydrolinalool	78–69-3	C10H22O	158.3	1414.3	685.32	1.2829
64	2,5-Dimethylpyrazine	123–32-0	C6H8N2	108.1	1335.9	542.84	1.1249
65	Anisyl formate	122–91-8	C9H10O3	166.2	1327.9	530.1	1.0889
66	Benzyl butanoate	103–37-7	C11H14O2	178.2	1336.3	543.5	1.4147
67	2-Methylpyrazine	109–08-0	C5H6N2	94.1	1245.8	419.84	1.3887
68	Ethyl 2-methyl-1,3-dioxolane-2-acetate	6413-10-1	C8H14O4	174.2	1160.5	341.43	1.2624
69	4-Methyl-2-pentanol	108–11-2	C6H14O	102.2	1183	356.1	1.2755
70	Ethylbenzene	100–41-4	C8H10	106.2	1159.9	341.04	1.4567
71	1-Pentanol	71–41-0	C5H12O	88.1	1274.1	453.7	1.2613
72	Linalyl acetate	115–95-7	C12H20O2	196.3	1259.8	436.29	1.6527
73	2-Heptenoic acid	18,999–28-5	C7H12O2	128.2	1189.4	360.43	1.4262
74	3-Methyl-1-butanol	123–51-3	C5H12O	88.1	1184.2	356.91	1.4837
75	Heptanal	111–71-7	C7H14O	114.2	1173.1	349.59	1.3164
76	Beta-Pinene	127–91-3	C10H16	136.2	1123.8	318.8	1.2244
77	2,3-Pentanedione	600–14-6	C5H8O2	100.1	1087.2	298.69	1.2932
78	2-Octanone	111–13-7	C8H16O	128.2	1296.1	482.33	1.3569
79	l-Carvone	2244-16-8	C10H14O	150.2	1257.5	433.56	1.8089
80	Sesamol	533–31-3	C7H6O3	138.1	1340.9	550.95	1.1856
81	Methyl caprylate	111–11-5	C9H18O2	158.2	1397.7	652.34	1.4079
82	(Z)-3-hexenyl butyrate	16,491–36-4	C10H18O2	170.3	1445.7	752.3	1.4207
83	3-Nonanone	925–78-0	C9H18O	142.2	1360.3	583.74	1.379
84	3-Pentanol	584–02-1	C5H12O	88.1	1097.3	303.4	1.1972
85	Methyl butanoate	623–42-7	C5H10O2	102.1	986.6	253.38	1.1514
86	Cis-3-hexenyl acetate	3681-71-8	C8H14O2	142.2	1335.3	541.85	1.3114
87	1-Hexanol	111–27-3	C6H14O	102.2	1347	561.04	1.3294
88	2-Nonanone	821–55–6	C9H18O	142.2	1389.9	637.33	1.4237
89	Heptanol	53,535–33-4	C7H16O	116.2	1459.4	783.6	1.3819
90	2-Hexanol	626–93-7	C6H14O	102.20	1295.20	480.96	1.57

Note: CAS: Chemical abstracts service; MW: Molecular weight; RI: Retention index; RT: retention time; DT: drift time.

In addition, the Upset plot (Fig. 2E) analyzed the intersection patterns of the VOCs detected in different treatment groups during storage (Song et al., 2024), revealing the differences in the diversity and dynamic differentiation of VOCs composition among the groups. This study identified 26 core VOCs that were commonly detected in all 18 samples, constituting the inherent basic flavor chemical background of WEL. The number of VOCs in the untreated WEL group significantly increased with storage time: from 40 at the initial stage (0 days) to 68 at day 36, an increase of 70.00%, reflecting the deep chemical and biological degradation of the egg yolk matrix under the combined action of microorganisms and endogenous enzymes (Fischer et al., 2022; Song et al., 2025). Notably, from the middle to late storage period (starting from day 20), multiple VOCs subsets were only present in the untreated WEL group and the PAS group, but not in the PEF treatment group. This specific absence pattern indicated that although PAS can achieve initial microbial inactivation, it fails to effectively inhibit the revival of spoilage bacteria and the generation of VOCs in the middle to late storage period, a phenomenon highly consistent with the significant rebound of microbial counts from day 12 to 20. In contrast, PEF treatment demonstrated an efficient blocking ability against the formation of VOCs related to late-stage deterioration. The minor changes in VOCs composition observed in the PEF group were mainly concentrated in the early storage period, and their component characteristics were related to the transient physical and chemical responses induced by the electric field (Dong, Ma, et al., 2026). These structural distribution results suggested that the PEF treatment group has the slowest VOCs profile succession rate, providing strong evidence for its superior ability to specifically inhibit flavor deterioration pathways.

The dynamic changes in the relative contents of various VOCs provided a deep-level analytical basis for the flavor deterioration process of WEL (Fig. 2F). Aldehydes are characteristic products of the primary oxidation of lipids and are the main contributors to the “rancid odor” (Dong et al., 2024). Dong, Ma, et al. (2026) pointed out that (Z)-4-Decenal, (E)-2-nonenal, and n-Pentanal have irritating and unpleasant odor and are often accompanied by fishy odors. As shown in Fig. 2D, the above-mentioned aldehydes in WEL group showed a continuous upward trend with storage time, while the corresponding signal intensities in the PEF group remained at the lowest level, significantly lower than those in the WEL group and the PAS group. The relative content of total aldehydes shown in Fig. 2F further confirmed that the PAS group had the highest increase, which was significantly higher than that in the WEL group and the PEF group, indicating that the PAS treatment not only failed to inhibit the generation of aldehydes but also possibly intensified lipid degradation due to heat-induced oxidation, leading to the enrichment of aldehydes. However, PEF interfered with the chain reaction of lipid oxidation, significantly weakening the free radical oxidation cleavage of unsaturated fatty acids, thereby reducing the production of these off-flavor substances from the source (Zhou et al., 2025) As the key enzyme catalyzing this process, the activity of lipoxygenase may be partially inactivated due to the conformational changes of proteins induced by PEF, which explains the low accumulation rate of aldehydes in the PEF group from the early stage of storage (Dong, Ma, et al., 2026). Ketones, as secondary oxidation products of lipids, are also a key source of off-flavors (Ren et al., 2022). The contents of 6-methyl-5-hepten-2-one, 3-hexanone, 2-nonanone and 2-octanone in the WEL group were significantly higher than those in the PAS and PEF groups, and the signal intensity of the PEF group remained consistently lower than that of the PAS group, further demonstrating that PEF treatment effectively inhibited the accumulation of these carbonyl compounds at the end stage. Alcohols are characteristic products of microbial metabolism. 1-Octen-3-ol is produced by microbial enzymatic oxidation of unsaturated fatty acids and is a typical “earthy” odor (Dong, Gao, et al., 2026; Han et al., 2025). This compound was detectable in the WEL group on the 4th day of storage and reached its peak on the 12th day. It was not detected in the PAS group until the 20th day. While in the PEF group, it remained at an extremely low level throughout the 36-day storage period, indicating that PEF has a long-lasting inhibitory effect on the metabolic activity of spoilage microorganisms. It is worth noting that the relative abundance of acids in the WEL group and the PAS group showed an explosive increase at the end of storage (day 36), which was a clear chemical indicator of intense microbial fermentation and rancidity (Rios-Corripio et al., 2022), directly resulting in a significant sour taste and spoilage sensory attributes of the egg liquid. In addition, sulfur/nitrogen-containing VOCs were significantly enriched in the WEL group in the later stage, and their generation was highly related to protein hydrolysis and microbial deamination/desulfurization metabolism, which were the core causes of the “rotten egg smell” and “ammoniacal odor” (Chen et al., 2025) In contrast, the PEF group successfully suppressed the excessive accumulation of the above acidic and nitrogen-containing spoilage markers at the end of storage. These results strongly confirmed that PEF treatment effectively maintained the proportion balance of various VOCs categories, thereby preserving the flavor quality of egg liquid.

Esters and terpenes are the main contributors to the fresh and pleasant background aroma of WEL. Although trace amounts of natural esters can impart fruity characteristics, the abnormal enrichment in the later stage was actually the result of enzymatic esterification reactions carried out by spoilage microorganisms using lipid oxidation products, generating volatile esters that can easily induce solvent-like or cloying-off odors (Zhang et al., 2018). Fig. 2F showed that the relative content of esters in the WEL group and the PAS group significantly increased in the later stage of storage, while the PEF group maintained a low and stable level of esters throughout the 36-day storage period. The results indicated that PEF effectively blocks this secondary off-flavor pathway by inhibiting microbial proliferation and the activity of key esterification enzymes from the source. This conclusion is also supported by the study of Bai et al. (2021), which reported that after PEF pretreatment of rice, the content of aldehydes decreased while the ester VOCs significantly increased, confirming that PEF can regulate the metabolic balance of flavor substances. Terpenoids not only contribute their own floral or citrus aromas but also have a sensory synergistic effect of masking unpleasant odors (Dong, Ma, et al., 2026). The PEF group maintained the natural terpene components such as Limonene throughout the storage period. In contrast, the terpene signal intensity in the PAS group continuously declined from the 12th day, and the residual aroma contribution was largely masked or interfered by a large amount of aldehydes, acids, and sulfur-containing spoilage VOCs. The findings of Conde et al. (2024) in the cassava flour further confirmed that PEF treatment can significantly increase the abundance of terpene VOCs and positively correlate with the improvement of sensory scores. The advantage of PEF in retaining heat-sensitive furan substances further confirms its mild regulatory ability on the transformation path of flavor precursors due to its non-thermal processing characteristics. Such compounds, as key intermediates in the Maillard reaction, mainly contribute positive flavor attributes such as caramel aroma, nutty aroma and roasting aroma in egg liquid systems (Dong, Gao, et al., 2026). In conclusion, PEF treatment effectively inhibited the generation of off-flavor substances during storage and significantly retains characteristic aroma components, demonstrating a better protective effect on the flavor quality stability of WEL than traditional thermal sterilization techniques.

### 3.3 Multivariate statistical analysis and screening of key flavor regulatory targets

To deeply explore the complex flavor data and visualize the dynamic evolution trajectory of the samples, we constructed orthogonal partial least squares discriminant analysis (OPLS-DA) models for each treatment group (Fig. 3A). As a supervised multivariate statistical method, OPLS-DA can effectively separate the characteristic variables between groups and reveal the intrinsic correlations among samples (Song et al., 2025). All models demonstrated excellent robustness: both R^2^ and Q^2^ values were close to 1.0, indicating a high degree of model fit and reliable predictive ability (Dong, Gao, et al., 2026). The score plots showed distinct migration patterns dependent on storage time in all groups, and the differences in spatial distribution visually confirmed the patterns discussed earlier. Among them, the WEL and PAS groups exhibited significant migration distances along the principal component axes, reflecting intense and continuous biochemical deterioration. In contrast, the spatial distribution of PEF-treated samples remained relatively concentrated in the early and middle stages, further confirming that PEF intervention enhanced the physical and chemical stability of the egg liquid matrix.

**Fig. 3:**
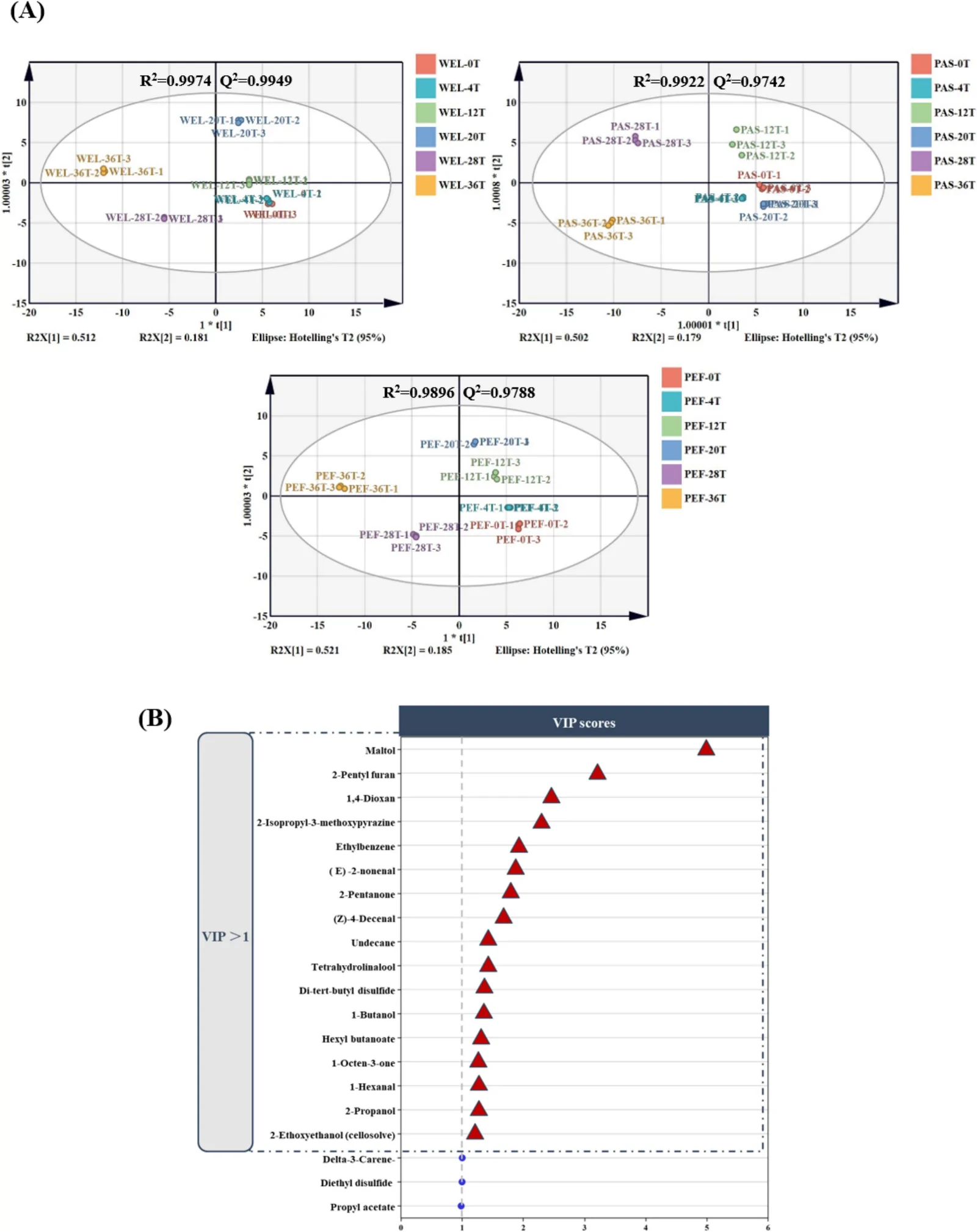

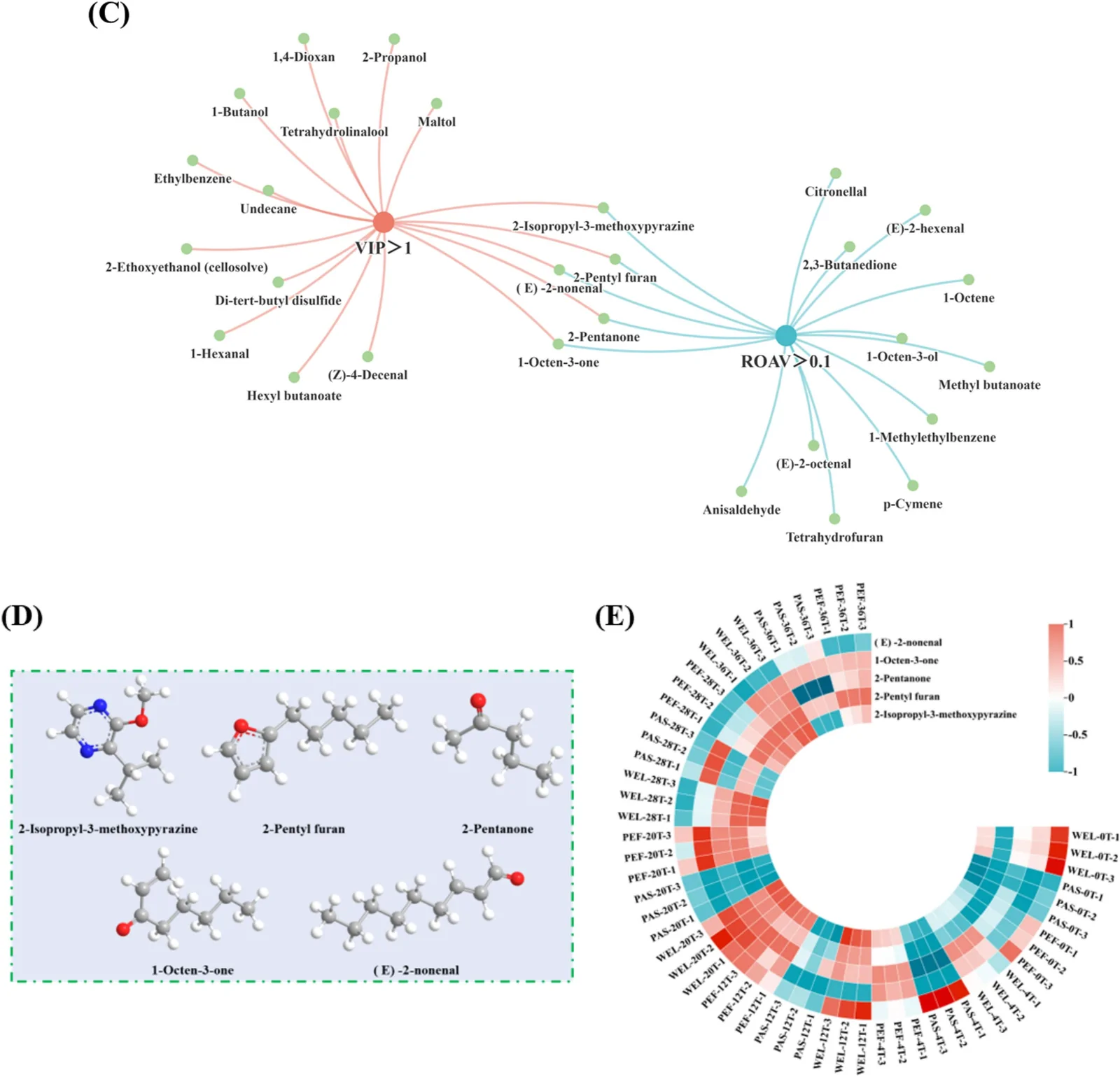
Multivariate statistical analysis of flavor substances in WEL during storage under different treatment methods. (A) Orthogonal partial least squares discriminant analysis (OPLS-DA) plot; (B) Variable importance in projection (VIP) value plot; (C) Screening of key flavor regulatory targets; (D) Chemical structures of key flavor compounds; (E) Heat map of key flavor compounds.

To further identify the key VOCs driving the separation between groups, this study employed variable importance in projection (VIP) analysis. Variables with a score higher than 1.0 were considered significant key feature markers. Based on the widely adopted screening threshold in the field of flavoromics (VIP > 1.0), variables with scores above this threshold were defined as having statistical significance and biological interpretability (Zhang et al., 2018). As shown in Fig. 3B, a total of 17 VOCs that significantly drove the statistical separation between samples were identified in this study. These include Maltol, 2-Pentyl furan, 1,4-Dioxan, 2-Isopropyl-3-methoxypyrazine, Ethylbenzene, (E)-2-nonenal, 2-Pentanone, (Z)-4-Decenal, Undecane, Tetrahydrolinalool, Di-tert-butyl disulfide, 1-Butanol, Hexyl butanoate, 1-Octen-3-one, 1-Hexanal, 2-Propanol, and 2-Ethoxyethanol (cellosolve).

To ensure that the selected compounds not only have statistical significance but also have practical sensory contribution significance, this study further combined the relative odor activity value (ROAV) for weighted screening. This strategy can effectively eliminate compounds with high content but weak sensory contribution, focusing on the key active components that truly dominate the overall flavor profile (Bi et al., 2025). Wang et al. (2026) reported that VOCs with ROAV ≥0.1 play an important regulatory role. As shown in Fig. 3C, a total of 16 VOCs with high sensory contribution were identified, mainly including 2-Isopropyl-3-methoxypyrazine, 1-Octen-3-one, (E)-2-nonenal, Anisaldehyde, 2,3-Butanedione, Citronellal, 1-Octen-3-ol, (E)-2-octenal, (E)-2-hexenal, Tetrahydrofuran, 1-Octene, p-Cymene, 2-Pentyl furan, 1-Methylethylbenzene, Methyl butanoate, and 2-Pentanone. Through the intersection analysis of the above dual screening strategy (VIP > 1.0 and ROAV ≥0.1) (Fig. 3D), a total of 5 key flavor-regulating target VOCs were finally screened out, namely 2-Isopropyl-3-methoxypyrazine, 2-Pentyl furan, 2-Pentanone, 1-Octen-3-one, and (E)-2-nonenal, and their three-dimensional molecular structures are shown in Fig. 3D.

It is worth noting that aldehydes are generally regarded as key chemical markers of lipid oxidation in egg products (Ren et al., 2022). Previous studies have shown that (E)-2-nonenal has a strong pungent and fishy odor, and its increased content significantly intensifies the off-flavor characteristics of the samples (Dong, Ma, et al., 2026). As shown in Fig. 3E, the content of (E)-2-nonenal in the WEL group and PAS group was significantly higher than that in the PEF group, which was consistent with their higher degree of lipid oxidation. In addition, 1-Octen-3-one is described as having an “earthy, musty, fishy” odor, and its relative content in the PEF group remained at a relatively low level throughout the storage period. Pyrazine compounds (such as 2-Isopropyl-3-methoxypyrazine) usually present nutty, roasted or green pepper-like aromas and have a significant olfactory contribution at extremely low concentrations. However, excessive accumulation can easily induce unpleasant grassy or earthy flavors (Zhou et al., 2025). The results showed that the relative content of this compound in the PEF group and PAS group was lower than that in the WEL group, suggesting that PEF treatment helps to inhibit its excessive generation. 2-Pentyl furan and 2-Pentanone have typical fruity odor characteristics and are often positively associated with fresh and pleasant sensory experiences. Compared with the PAS group, the content changes of these two compounds in the PEF-treated samples were more stable, indicating that PEF technology is more conducive to maintaining the stability of pleasant flavor components in egg liquid.

### 3.4 The regulatory effects of different treatment methods on protein oxidation in WEL during storage

Free sulfhydryl groups and TVB-N are key indicators reflecting protein oxidation and degradation, directly associated with flavor deterioration in WEL (Yu et al., 2021; Zheng et al., 2025). As shown in Fig. 4A-B, pasteurization induced protein degradation due to thermal effects at the early stage of storage, resulting in significantly higher initial TVB-N levels compared to untreated and PEF treated group. As the storage period extends, the protein deterioration rate of the WEL group is the fastest. The loss rate of its free sulfhydryl groups reaches as high as 92.90% on the 36th day, and the TVB-N exceeds the spoilage threshold of 20 mg/100 g on the 4th day. Although the PAS group delayed the deterioration to a certain extent, the TVB-N exceeded the standard on the 20th day. In contrast, PEF treatment demonstrated superior protein structural stability. By the 36th day, the free sulfhydryl loss rate of the PEF group was only 36.53%, and the TVB-N exceeded the standard threshold. The excellent oxidative stability of the PEF group during the middle and late stages of storage is related to its passivation effect on the enzyme system and regulation of the protein structure (Rios-Corripio et al., 2022). PEF can inactivate endogenous proteases and pro-oxidant enzymes and induce conformational changes in proteins, thereby inhibiting protein oxidation (Dong et al., 2024). Notably, the destabilization and consumption of free sulfhydryl groups represent a critical prerequisite for the formation of sulfur-containing VOCs, while the accumulation of TVB-N directly leads to the generation of amine-related off-flavors (Liu et al., 2026). By inhibiting protein oxidation and enzymatic degradation, PEF effectively blocks the formation of odor precursors, thus significantly preserving the natural flavor characteristics of WEL.

**Fig. 4:**
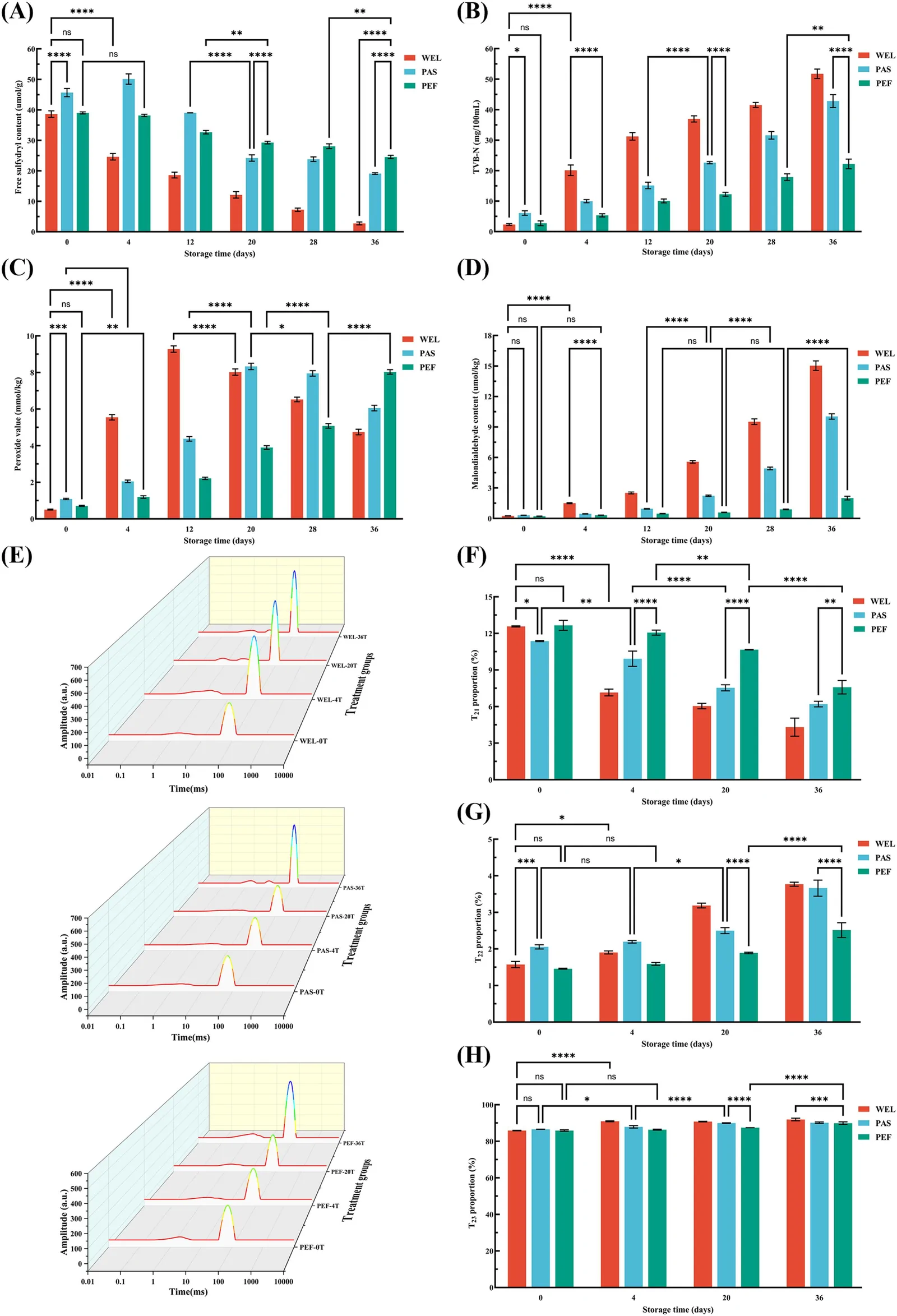
Regulatory effects of different treatments on protein, lipid oxidation and water migration of WEL during storage. (A) Free sulfhydryl content; (B) Total volatile basic nitrogen (TVB-N) value; (C) Peroxide value; (D) Malondialdehyde content; (E) Low-field nuclear magnetic resonance (LF-NMR) relaxation spectrum of water content; (F—H) Water content of T_21_, T_22_ and T_23_ components. “*” indicates the significance level of differences between groups: ****p < 0.0001, ***p < 0.001, **p < 0.01, *p < 0.05; NS indicates no significant difference between groups.

### 3.5 The regulatory effects of different treatment methods on lipid oxidation in WEL during storage

The POV and malondialdehyde content are the core indicators for evaluating lipid oxidation, which directly determine the formation of typical oxidative off-flavors such as rancid and metallic tastes in WEL (Dong, Ma, et al., 2026). As shown in Fig. 4C-D, the POV of the WEL and PAS group reached their peaks on the 12th and 20th days respectively and then decreased sharply during storage, indicating that a large amount of primary hydroperoxides were decomposed into volatile off-flavor substances such as aldehydes and ketones. Meanwhile, the malondialdehyde content in these two groups continued to increase with the extension of storage time. In contrast, the POV of the PEF group only showed a continuous and slow accumulation during the 36-day storage period, without an inflection point of attenuation and decomposition. At the same time, its TBARS value was always strongly suppressed at an extremely low level. This is attributed to the dual effects of PEF in efficiently inactivating microorganisms and endogenous pro-oxidant enzymes (Sun et al., 2024). Research reports that PEF treatment can disrupt the structure of lipoprotein complexes, possibly exposing peptide segments or amino acid residues with antioxidant activity, thereby quenching free radicals and delaying the initiation of the lipid peroxidation chain reaction (Zhou et al., 2024). PEF treatment showed a significant advantage over traditional thermal sterilization in inhibiting fat oxidation during the storage of WEL. The research of Sepulveda et al. (2009) on liquid milk also confirmed that PEF can significantly extend the oxidation induction period and maintain the oxidative stability of the product without inducing a thermal effect.

### 3.6 The regulatory effects of different treatment methods on water migration in WEL during storage

The moisture status directly affects the quality stability and shelf life of egg products (Song et al., 2024). Fig. 4E-H showed the changes in relative contents of bound water (T₂₁), immobilized water (T₂₂), and free water (T₂₃) in WEL from different treatment groups during storage. As storage time increased, the bound water content decreased in all three groups. WEL group dropped from 12.57% to 4.31%, PAS group from 11.37% to 6.20%, and PEF group from 12.66% to 7.58%. The immobilized water content showed an increasing trend. WEL group rose from 1.57% to 3.77%, PAS group from 2.06% to 3.66%, and PEF group from 1.46% to 2.51%. Free water content also increased. WEL group increased from 85.85% to 91.92%, PAS group from 86.57% to 90.14%, and PEF group from 85.88% to 89.90%. These results indicated a continuous decline in bound water accompanied by increases in both immobilized and free water. This suggested that the relaxation of the protein network structure in WEL during storage leads to reduced water-holding capacity. Differences in water migration during storage reveal the regulatory effect of PEF treatment on water distribution in WEL. The initial redistribution of water in PEF-treated samples may be related to the direct impact of electric fields on protein structures, whereas changes observed in pasteurized samples are attributed to heat-induced protein denaturation and aggregation (Song et al., 2024). The PEF group exhibited the smallest increase in free water and the least reduction in bound water, indicating its effectiveness in maintaining the integrity of the protein network and inhibiting the transition of water from bound to free states, thereby delaying quality deterioration (Sun et al., 2024). Sheng et al. (2024) found that higher bound water and lower free water levels help retain flavor precursors and suppress off-flavor formation. These findings corroborate previous conclusions, confirming that PEF treatment effectively stabilizes the spatial distribution of water in WEL by significantly delaying the dissociation of bound water and release of free water, providing a crucial physical basis for long-term quality preservation. Thus, PEF outperforms pasteurization in extending shelf life and maintaining flavor quality.

### 3.7 The effects of different treatment methods on microbial community in WEL during storage

Microbial metabolism is the core factor driving the degradation of WEL and the generation of off - odors (Wang et al., 2025). Alpha diversity can reflect the characteristics of the community structure. The ACE and Chao indices characterize species richness. The Shannon index measures diversity and evenness, with a higher value indicating a more complex and balanced community. The Simpson index reflects the dominance degree of dominant species, with a higher value indicating a more homogeneous community. The Pielou_e index quantifies the evenness of species distribution, and a value approaching 1.0 indicates a high degree of balance. A Coverage index greater than 0.99 indicates sufficient sequencing depth and reliable data (Vieira et al., 2019; Wang et al., 2024). As shown in Fig. 5A-F, the WEL group had the lowest ACE, Chao, and Shannon indices, the highest Simpson index, and the lowest Pielou_e index, indicating low microbial community richness, a highly simplified structure, and dominance by a few spoilage dominant bacteria (Lu et al., 2024). The PAS group had intermediate values for all indices, showing a moderate increase in diversity and evenness. The PEF group had the highest ACE, Chao, and Shannon indices, the lowest Simpson index, and the highest Pielou_e index, indicating a rich community of species, a balanced distribution, and no obvious dominant bacteria (Wang et al., 2025). These results confirmed that PEF treatment significantly inhibited initial spoilage bacteria and reconstructs a micro-ecological structure with low abundance and high evenness, blocking the explosive accumulation of off-odor metabolites at the source. In contrast, PAS group only partially enhances diversity, and its regulatory efficacy was weaker than that of PEF. In addition, the Coverage indices of all three groups of samples were higher than 0.99 (Fig. 5D), indicating that the 16S rRNA gene sequencing depth sufficiently covered the vast majority of microbial sequences in the samples (Lu et al., 2024). This indicated that the data was highly reliable and can support the aforementioned diverse analysis conclusions.

**Fig. 5:**
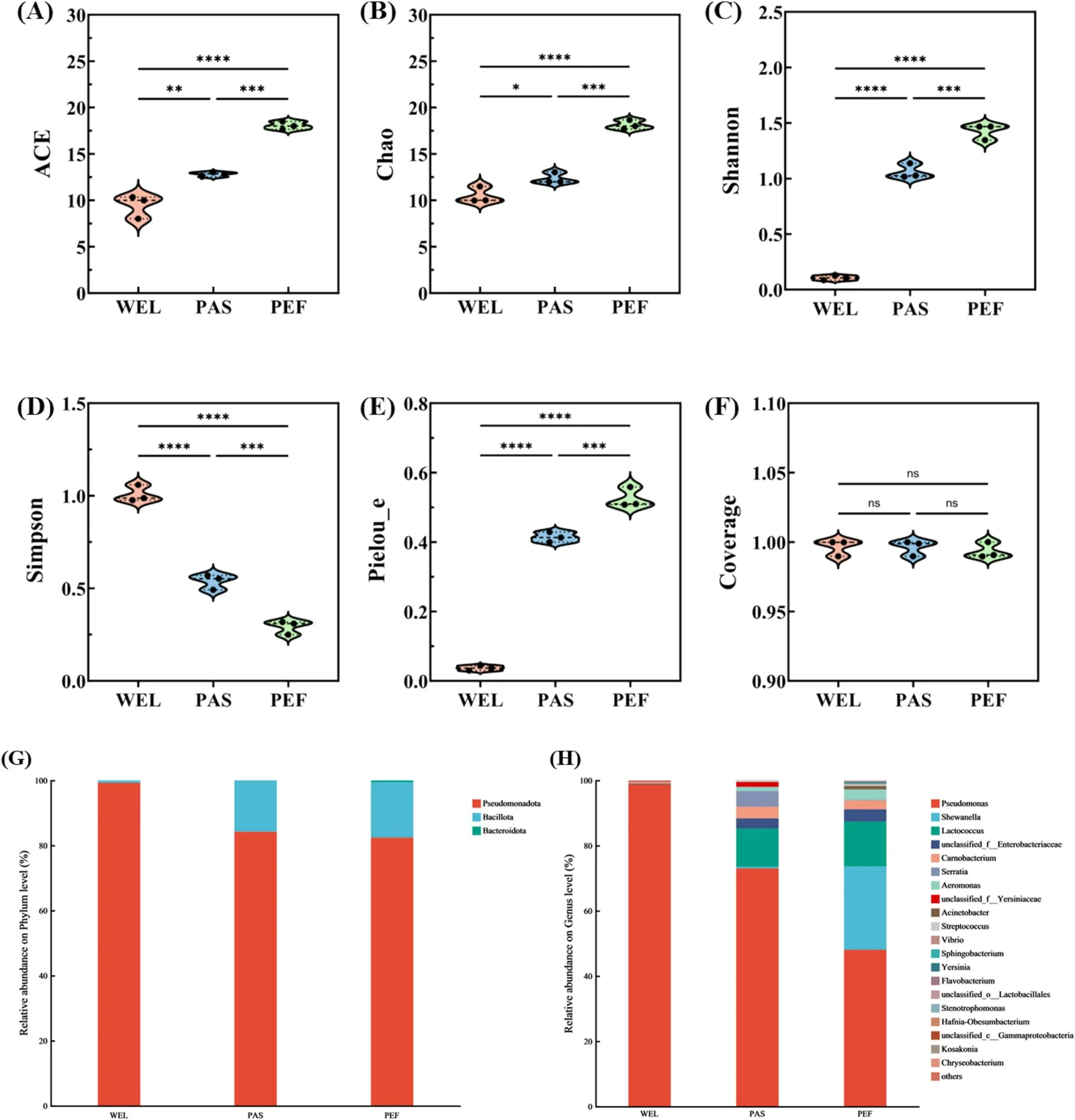

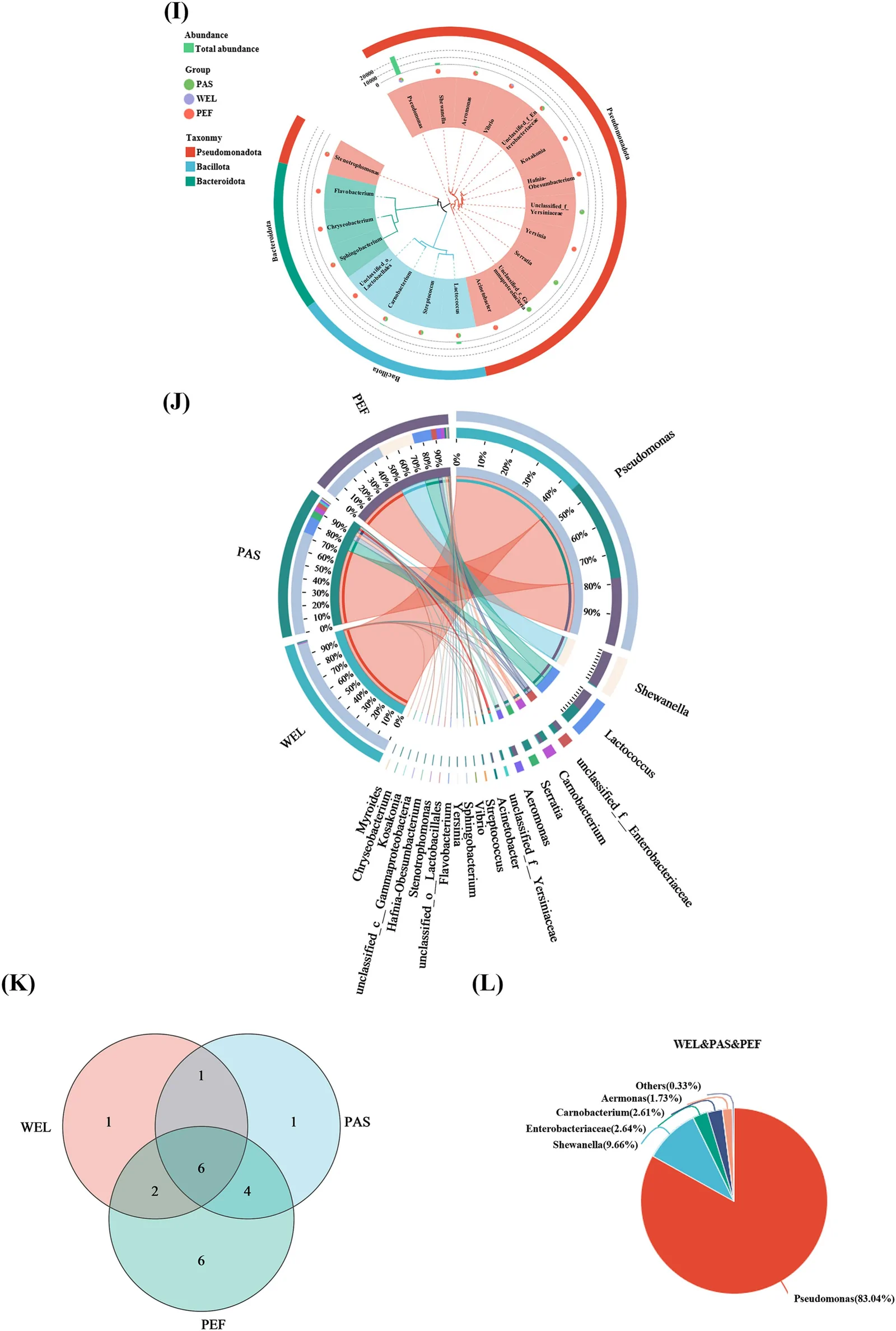
Analysis of microbial community structure at the end of storage (36 T) under different treatments. (A) ACE index; (B) Chao index; (C) Shannon index; (D) Simpson index; (E) Pielou_e index; (F) Coverage index; (G) Relative abundance of microorganisms at the phylum level; (H) Relative abundance of microorganisms at the genus level; (I) Circular evolutionary tree diagram; (J) Community circos diagram; (K) Species Venn diagram; (L) Species distribution pie chart. “*” indicates the significance level of differences between groups: ****p < 0.0001, ***p < 0.001, **p < 0.01, *p < 0.05; NS indicates no significant difference between groups.

Fig. 5G-H showed the relative abundance of microbial communities at both phylum and genus levels. At the phylum level, the untreated WEL group exhibited extremely high species homogeneity, with *Pseudomonadota* accounting for over 95% of the community, while *Bacillota* and *Bacteroidota* together comprised only 4.2%. After processing interventions, the community structure changed significantly. The proportion of *Pseudomonadota* decreased in both the PAS and PEF groups, whereas the relative abundance of *Bacillota* increased markedly. This evolutionary trend is highly consistent with Tan et al.'s (2025) findings in soy sauce flavor research, which demonstrated that processing methods can effectively break the dominance of originally prevalent phyla and drive the community toward more pressure resistant microbial populations. At the genus level, the WEL group was almost entirely dominated by *Pseudomonas*, a typical psychrotrophic spoilage bacterium known for its strong secretion of proteases and lipolytic enzymes, making it a primary source of off-odors such as fishy and rancid smells in egg products (Gram et al., 1990). In contrast, pasteurization and PEF treatments showed superior microbial inactivation effects, with PEF demonstrating greater suppression than pasteurization. In the PEF group, the abundance of *Pseudomonas* dropped dramatically from 99.0% to 48.2%, while the proportions of genera such as *Shewanella*, *Lactococcus*, *Enterobacteriaceae*, and *Carnobacterium* increased relatively. According to ecological competition theory, a highly diverse and balanced community structure can suppress the growth of specific spoilage bacteria through interspecies competition, thereby effectively slowing the accumulation of TVB-N and lipid oxidation chain reactions (Wang et al., 2024). Thus, PEF treatment efficiently targeted and reduced the dominance of core odor-causing genera like *Pseudomonas*, reshaping the microbial community toward greater diversity and balance, and thereby blocking the flavor deterioration pathway driven by single dominant spoilage microbes at the biological origin. The topology of the circular phylogenetic tree (Fig. 5I) revealed that the WEL group had the most extensive and widespread microbial branches, with dominant clades concentrated among multiple genera within the *Pseudomonadota* phylum, indicating high community complexity. In contrast, the tree of PEF group displayed a clear pruning effect, with several previously expanded spoilage related branches in the WEL group being effectively suppressed. Relative quantification of species abundance via community pie charts (Fig. 5J) revealed that PEF treatment not only reduced the relative abundance of *Pseudomonas*, but also induced balanced succession of genera such as *Shewanella*, *Lactococcus*, and *Carnobacterium*. By weakening the ecological dominance of *Pseudomonas*, PEF blocked the evolutionary pathway leading to egg white flavor deterioration.

To further identified the core microbial communities in WEL, the distribution characteristics of the common and unique microbial communities among different groups were analyzed. The results of the Venn diagram (Fig. 5K) showed that there were 6 phylum level microorganisms common to the three treatment groups of WEL, PAS, and PEF. Notably, the WEL group, PAS group, and PEF group possess 1, 1, and 6 unique phyla respectively. This confirmed that PEF not only effectively reduces the overall microbial load but also exerts a reconstructive effect on the original microbial flora by inducing the selective enrichment or survival of specific taxa. The pie chart of species distribution at the genus level (Fig. 5L) further revealed the pattern of dominant spoilage bacteria. *Pseudomonas* was the absolutely dominant group, with a relative abundance of 83.04%. Followed by *Shewanella*, *Enterobacteriaceae*, *Carnobacterium*, and *Aeromonas*, with relative abundances of 9.66%, 2.64%, 2.61%, and 1.73% respectively, and the remaining genera accounted for only 0.33% in total. This distribution characteristic was highly consistent with the results at the phylum level. As the core genus, the high proportion of *Pseudomonas* confirmed its significant tolerance to pasteurization, which might be attributed to its inherent low temperature adaptation mechanism and the repair ability after sublethal damage (Ding et al., 2024).

### 3.8 Multivariate correlation analysis

The quality deterioration of WEL during storage is associated with microbial metabolism, biochemical degradation, and moisture migration (Tan et al., 2025). To gain deeper insight into the mechanisms underlying this deterioration, Pearson correlation analysis and Mantel tests were employed to examine the relationships among microbial community succession, physicochemical changes, and flavor compounds (Fig. 6). Correlation analysis revealed that (E)-2-nonenal and 1-octen-3-one showed strong positive correlations with lipid oxidation indicators, confirming their origin as secondary products of lipid oxidation. This phenomenon arises from the peroxidation of unsaturated fatty acids via radical chain reactions during lipid oxidation, which subsequently degrade through β-scission pathways to form low-threshold, highly volatile aldehydes and ketones (Dong, Gao, et al., 2026). The contents of typical off-flavor compounds such as 2-isopropyl-3-methoxy pyrazine were significantly positively correlated with the relative abundances of *Pseudomonas* and *Shewanella* genera (*r* > 0.5, *p* < 0.01). Whereas VOCs with grassy or fruity notes, such as 2-pentanone, exhibited significant negative correlations (*r* > 0.5, *p* < 0.05), indicating that these two bacterial genera drive spoilage processes and impair the inherent flavor quality of WEL (Lu et al., 2024). Mantel tests further revealed significant associations between environmental factors and microbial community structures (*R* > 0.5, *P* < 0.05). Results indicated that both *Pseudomonas* and *Shewanella* showed positive correlations with quality deterioration indices, confirming their roles as key functional taxa driving multidimensional quality decline during refrigerated storage of WEL.

**Fig. 6:**
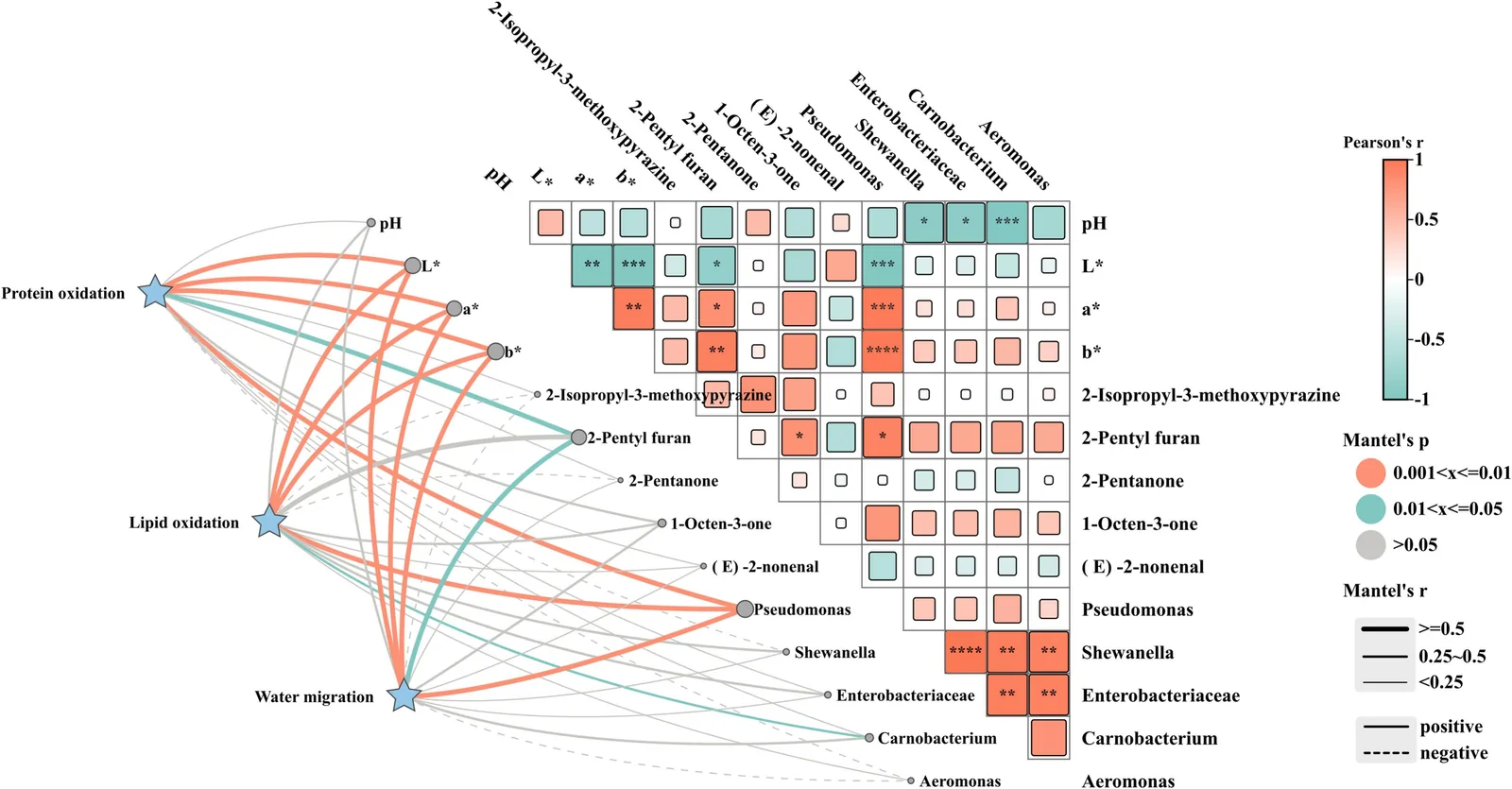
Correlation analysis of WEL quality properties, key flavor compounds, microbial communities, and processes such as protein oxidation, lipid oxidation, and water migration during storage.

From a physicochemical perspective, these two genera exhibited strong positive correlations with a^⁎^ and b^⁎^ values. This was consistent with the ability of *Pseudomonas* to secrete extracellular enzyme systems that accelerate the degradation of egg components, thereby causing color shifts (Dong et al., 2024). Additionally, Mantel test results demonstrated significant correlations between color changes and the oxidation of proteins and lipids. Pointed out that lipid oxidation products can polymerize to form brown oxidized polymers, resulting in darker product color. Song et al. (2025) reported that oxidative degradation of lipids leads to polymerization of secondary oxidation products, yielding brown-colored macromolecular aggregates and consequent color darkening. Similarly, Ouyang et al. (2023) observed progressive browning during oxidative modification of whey protein isolate, underscoring the critical role of protein oxidation in chromatic deterioration. The effects of lipid and protein oxidation on color reported in the literature are similar to our findings. On the other hand, pH as a key indicator reflecting the acid-base balance of the system, showed a negative correlation with most quality degradation parameters. This suggested that as storage time increased, organic acids produced by microbial metabolism and volatile fatty acids generated from lipid oxidation gradually accumulate, leading to environmental acidification and quality decline (Han et al., 2025). The continuous decrease in pH may further alter the charge state and conformational stability of proteins, thereby accelerating protein oxidation reactions and affecting the flavor quality of WEL (Dong et al., 2024).

The correlation analysis revealed the complete chain of egg liquid quality deterioration. Dominant spoilage bacteria represented by *Pseudomonas* initiated the oxidation of proteins and lipids through metabolic activities, inducing the accumulation of characteristic off-flavor components and changes in the water distribution state. The non-enzymatic browning pathway led to color deterioration, which constituted the core driving network for the quality deterioration of WEL during storage. PEF treatment effectively disrupted core driving factors such as *Pseudomonas* and *Shewanella* at the biological level and simultaneously inhibited the oxidative chain reaction and water loss, thereby maintaining the flavor stability of WEL.

## 4 Conclusions

This study revealed the key mechanism by which PEF treatment suppresses flavor deterioration in WEL through targeted regulation of chemical oxidation and microbial succession. PEF treatment extends the microbial shelf life of WEL to 36 days, which was significantly superior to the 20-day shelf life achieved by pasteurization. Moreover, the minimal fluctuations in pH value and chromaticity confirmed its long term inhibitory effect on microorganisms. Based on GC-IMS technology, a total of 90 VOCs were identified in stored WEL. Using a combined VIP and ROAV screening strategy, five critical flavor regulating targets were identified: 2-isopropyl-3-methoxy pyrazine, 2-pentylfuran, 2-pentanone, 1-octen-3-one, and (E)-2-nonenal. Results showed that PEF treatment significantly suppressed the accumulation of characteristic deteriorative VOCs during mid-to-late storage, including aldehydes and ketones associated with rancidity such as (E)-2-nonenal and 1-octen-3-one, sulfur- or nitrogen-containing off-flavor compounds like diethyl disulfide and 2-isopropyl-3-methoxy pyrazine, as well as esters responsible for unpleasant fruity and solvent-like odors. Protein and lipid oxidation indicators revealed that the rate of free thiol loss in the PEF group was significantly lower than in the WEL and PAS groups. TVB-N levels remained within acceptable limits throughout storage, while POV and TBARS values consistently stayed at the lowest levels. This confirmed that PEF effectively blocked oxidative degradation pathways from the source by deactivating endogenous pro-oxidative enzymes and interrupting radical chain reactions, thereby preventing the formation of off-flavors. Low-field nuclear magnetic resonance analysis indicated that the PEF group exhibited the smallest reduction in bound water and the smallest increase in free water, effectively maintaining the integrity of the protein network structure, thus enhancing the matrix's ability to retain moisture and flavor precursors and slowing down the kinetics of quality deterioration. High-throughput 16S rRNA sequencing revealed that PEF treatment drastically reduced the relative abundance of *Pseudomonas* from 99.0% to 48.2%, and induced community restructuring toward greater diversity and balance, biologically disrupting the flavor deterioration pathway driven by dominant spoilage bacteria. Pearson and Mantel correlation analyses further confirmed that *Pseudomonas* and *Shewanella* were core genera driving quality deterioration, showing significant positive correlations with lipid oxidation, color degradation, and characteristic off-flavor compounds, whereas PEF treatment effectively severed these key drivers. As a non-thermal processing technology, PEF demonstrates superior potential over conventional pasteurization in high-quality liquid egg processing, providing both theoretical foundation and technical support for extending the shelf life of WEL and preserving its natural flavor and quality.

## CRediT authorship contribution statement

**Liu Dong:** Writing – original draft, Methodology, Investigation, Formal analysis, Data curation. **Enhui Ma:** Methodology, Investigation, Formal analysis. **Renrui Zheng:** Methodology, Investigation. **Jun Jiang:** Software, Investigation. **Xinqing Lu:** Methodology, Conceptualization. **Lili Zhang:** Visualization, Supervision. **Songyi Lin:** Writing – review & editing, Validation, Project administration, Funding acquisition, Conceptualization.

## Declaration of competing interest

The authors declare that they have no known competing financial interests or personal relationships that could have appeared to influence the work reported in this paper.

## Data Availability

Data will be made available on request.
